# Pre‐Existing Th1 Immunity Outperforms Age in Predicting Antibody Responses to SARS‐CoV‐2 Inactivated Vaccines

**DOI:** 10.1002/advs.202514147

**Published:** 2025-11-16

**Authors:** Chanyuan Ye, Xiaoli Zhang, Lingfeng Qiu, Jia Ji, Jueqing Gu, Hongyu Jia, Yingfeng Lu, Jiyang Chen, Zelu Meng, Jiangshan Lian, Hangping Yao, Xun Zeng, Yida Yang

**Affiliations:** ^1^ State Key Laboratory for Diagnosis and Treatment of Infectious Diseases National Clinical Research Center for Infectious Diseases National Medical Center for Infectious Diseases Collaborative Innovation Center for Diagnosis and Treatment of Infectious Diseases Department of Infectious Diseases The First Affiliated Hospital Zhejiang University School of Medicine Hangzhou 310024 China; ^2^ Jinan Microecological Biomedicine Shandong Laboratory Jinan 250117 China

**Keywords:** aged, CD4^+^T, inactivated vaccine, SARS‐CoV‐2, Th1 cells

## Abstract

Understanding and enhancing vaccine‐induced immune responses in the elderly population is critical, as they face elevated risks of severe COVID‐19. This work systematically delineates age‐associated alterations in innate and adaptive immunity and their impact on responses to SARS‐CoV‐2 inactivated vaccination. Compared to young mice, aged mice exhibited polarized bystander Th1 CD4^+^T cell populations (31.68 ± 5.62% versus 3.31 ± 0.48%) with distinct transcriptomic signatures, which augmented humoral immunity in aged mice. In human cohorts, post‐vaccination antigen‐specific antibody titers are comparable across different age groups. Stratified analysis based on antibody concentration reveals that high‐responder individuals possess elevated pre‐existing Th1 cells at baseline, which exhibit a modest positive correlation with post‐vaccination antibody titers (r = 0.423, p = 0.001). Therefore, compared to age‐based stratification, baseline Th1 cells serve as a superior predictive biomarker for antibody generation following SARS‐CoV‐2 inactivated vaccination. Collectively, these findings unveil novel mechanisms underlying Th1‐mediated vaccine immunogenicity, offering pivotal insights for developing next‐generation vaccines with optimized protective efficacy.

## Graphical abstract

Challenging the focus on age, this study finds pre‐existing T helper 1 cells are the key biomarker for predicting antibody response to SARS‐CoV‐2 inactivated vaccines. In both mice and humans, high responders exhibited elevated baseline T helper 1 cells, which correlated with robust humoral immunity, offering crucial guidance for improving vaccine design.

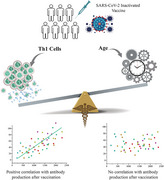

## 1 Introduction

Vaccination represents the most cost‐effective life‐saving medical intervention, playing a pivotal role in preventing both established and emerging infectious diseases.^[^ ^1^ ^]^ Although currently available COVID‐19 vaccines substantially mitigate risks of severe disease, hospitalization, and mortality, booster vaccinations have demonstrated limited efficacy in halting viral transmission.^[^ ^2^ ^]^ The persistent circulation of SARS‐CoV‐2 facilitates the emergence of variant strains with enhanced capacity to evade pre‐existing vaccine‐induced immunity,^[^ ^3^ ^]^ thereby underscoring the development of novel universal vaccines capable of eliciting broad‐spectrum protection and durable immune responses as a critical research priority in the COVID‐19 vaccine landscape.

Owing to the exigency of early COVID‐19 vaccine development, SARS‐CoV‐2 inactivated vaccines emerged as the most extensively administered vaccine platform across China.^[^ ^4^ ^]^ While numerous studies^[^ ^5^, ^6^ ^]^ have substantiated their efficacy, waning humoral immunity over time has raised concerns regarding the durability of immunological memory post‐vaccination. Multiple determinants, including host genetics and environmental factors such as age, biological sex, dietary patterns, geographical location, microbiome composition, and metabolite profiles, collectively shape innate and adaptive immune responses to vaccination.^[^ ^7^ ^]^ Age‐dependent immune remodeling, termed immunosenescence, is widely implicated in heightened susceptibility to infection and diminished vaccine‐induced protection.^[^ ^8^ ^]^ This phenomenon is principally attributed to compromised B and T cell functionality in elderly individuals, culminating in quantitatively and qualitatively impaired immune responses to pathogens and vaccines relative to younger subjects.^[^ ^9^, ^10^ ^]^ Current approaches to evaluating vaccine‐induced immune responses frequently rely on demographic parameters such as chronological age, which, while accessible, often fail to capture the complex interplay of immunological factors determining individual vaccine efficacy. Recent advances have sought to identify more precise biomarkers—such as pre‐existing antigen‐specific T cells, transcriptional signatures, or metabolomic profiles—to predict vaccine responsiveness.^[^ ^11^, ^12^ ^]^ However, these strategies have yet to be systematically validated in the context of SARS‐CoV‐2 inactivated vaccines, particularly across diverse age groups experiencing immunosenescence. Notably, emerging evidence reveals paradoxical age‐related immune patterns. A recent study demonstrated universal seroconversion following SARS‐CoV‐2 infection, yet observed significantly elevated antibody titers in elderly patients, conferring superior protection compared to those under 50 years.^[^ ^13^ ^]^ A similar report showed higher neutralizing antibody titers in convalescent elderly individuals versus younger counterparts.^[^ ^14^ ^]^ Yang et al. further corroborated an age‐dependent positive correlation between SARS‐CoV‐2 antibody levels post‐infection across adulthood.^[^ ^15^ ^]^ Conversely, other studies detected no significant inter‐age‐group disparities in antibody titers following COVID‐19 vaccination among healthy populations.^[^ ^16^ ^]^ The mechanistic underpinnings of these discordant observations remain elusive. Elucidating the precise mechanisms governing age‐related dysregulation of immune responses is consequently imperative for developing next‐generation vaccines with enhanced protective efficacy.

In this study, we comprehensively delineated the impact of immunosenescence on SARS‐CoV‐2 inactivated vaccination through an integrated approach combining human cohort analysis with murine models. Although aging demonstrably influences protective antibody production kinetics,^[^ ^17^ ^]^ we observed substantial inter‐individual heterogeneity, underscoring the critical contribution of environmental and host‐intrinsic variables beyond chronological age. Given that immunological status is modulated by multifactorial determinants, stratifying vaccine protective efficacy solely by age introduces significant bias. In contrast to conventional age‐based stratification, we identify baseline Th1 cellular immunity as a more reliable predictor of humoral response. Notably, baseline Th1 cells exhibited a more robust correlation with post‐vaccination antibody titers than age itself. These findings not only provide a novel mechanistic insight into the variability of vaccine responses in aging populations but also propose a translatable biomarker for optimizing vaccination strategies. By shifting the focus from chronological age to functional immune status, our work supports the development of personalized vaccination frameworks and enhances the clinical potential of existing inactivated vaccines, particularly in older adults.

## 2 Result

### 2.1 Age‐Associated Enhancement of Humoral Immunity Following SARS‐CoV‐2 Inactivated Vaccination

**Figure 1:**
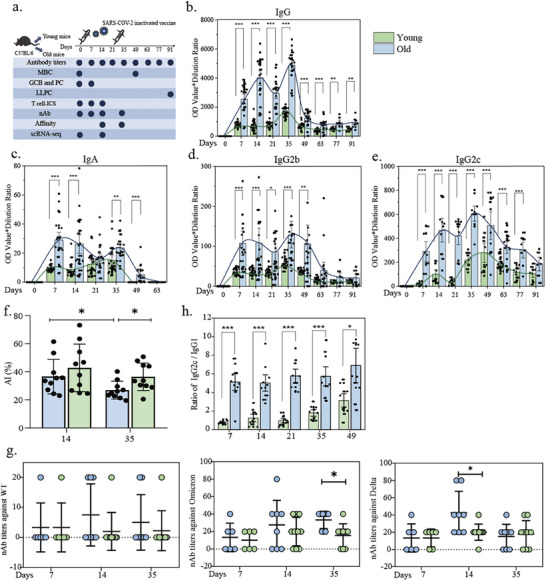
Characteristics of antibodies produced in aged and young mice after SARS‐CoV‐2 inactivated vaccination. a) Schematic diagram of experimental process. b–e) Comparison of IgG, IgA, IgG2b, and IgG2c levels with time in the serum of aged and young mice after SARS‐CoV‐2 inactivated vaccination. Data were normalized by subtracting the background signal from age‐matched naïve controls. f) Comparison of affinity indices (AI) of antibodies produced in the serum of aged and young mice at D14 after the first and the second dose of SARS‐CoV‐2 inactivated vaccination. g) Levels of neutralizing antibodies produced against different COVID‐19 variants in aged and young mice at different time points after SARS‐CoV‐2 inactivated vaccination. h) Ratio of IgG2c to IgG1 produced in the serum of aged and young mice after SARS‐CoV‐2 inactivated vaccination.

To investigate the age‐related differences in immune responses to SARS‐CoV‐2 inactivated vaccination, we established a murine aging model using C57BL/6 mice, which were categorized into two groups: aged mice (16‐24 months) and young mice (6–8 weeks). Mice received primary (Day 0) and booster (Day 21) immunizations with SARS‐CoV‐2 inactivated vaccine, with Blood/tissue samples collected at the designed time points for quantification of S1‐specific antibodies and analysis of corresponding immunological indicators (**Figure** **1**a). As antibody‐mediated protection is governed by both quantity and affinity,^[^ ^18^ ^]^ we interrogated the differences in amounts and affinities of S1‐specific antibodies in both groups, respectively. We found that the serum level of S1‐specific antibodies (total IgG, IgA, and IgM) was significantly higher in aged mice than that in young mice regardless of primary or secondary vaccination (Figure 1b,c, Figure S1a, Supporting Information). Subclass analysis revealed that S1‐specific IgG2b, IgG2c (but not IgG1/IgG3) exhibited significantly and persistently higher levels in aged mice compared to young mice (Figure 1d,e, Figure S1b,c, Supporting Information). However, although aged mice generated more high‐affinity IgG (Figure S1d, Supporting Information), their antibody affinity index (AI), the ratio of the high‐affinity IgG to total IgG, matched young mice post‐primary immunization but declined post‐secondary immunization (Figure 1f). This suggested that the substantial increase in S1‐specific antibodies in aged mice after the second vaccination was low‐affinity antibodies. The inability of secondary vaccination to induce a higher proportion of high‐affinity antibodies in both aged and young mice potentially explained the limited duration of protection afforded by two doses of the SARS‐CoV‐2 inactivated vaccine. Additionally, the evaluation of neutralizing antibodies (NAbs) serves as a critical indicator for evaluating vaccine efficacy.^[^ ^19^ ^]^ NAbs play a fundamental role in immune protection by preventing SARS‐CoV‐2 infection, thereby conferring long‐term immunity.^[^ ^20^ ^]^ To explore the immunogenicity profile of SARS‐CoV‐2 inactivated vaccine in aged and young mice, we systematically measured the NAbs titers against different SARS‐CoV‐2 variants in serums at multiple time points post‐vaccination and found that NAbs titers against SARS‐CoV‐2 variants were comparable between groups (Figure S1e–g, Supporting Information), with aged mice having transiently higher titers against Omicron (day 35) and Delta (day 14) than young mice (Figure 1g, Figure S1h, Supporting Information). The IgG2c/IgG1 ratio is used to evaluate the bias of immune response toward Th1 or Th2 pathways following vaccination.^[^ ^21^ ^]^ Our findings revealed that the elevated IgG2c/IgG1 ratio in aged mice indicated a Th1‐polarized immunity (Figure 1h).

### 2.2 Generalizability of Enhanced Antibody Responses in Aged Mice

**Figure 2:**
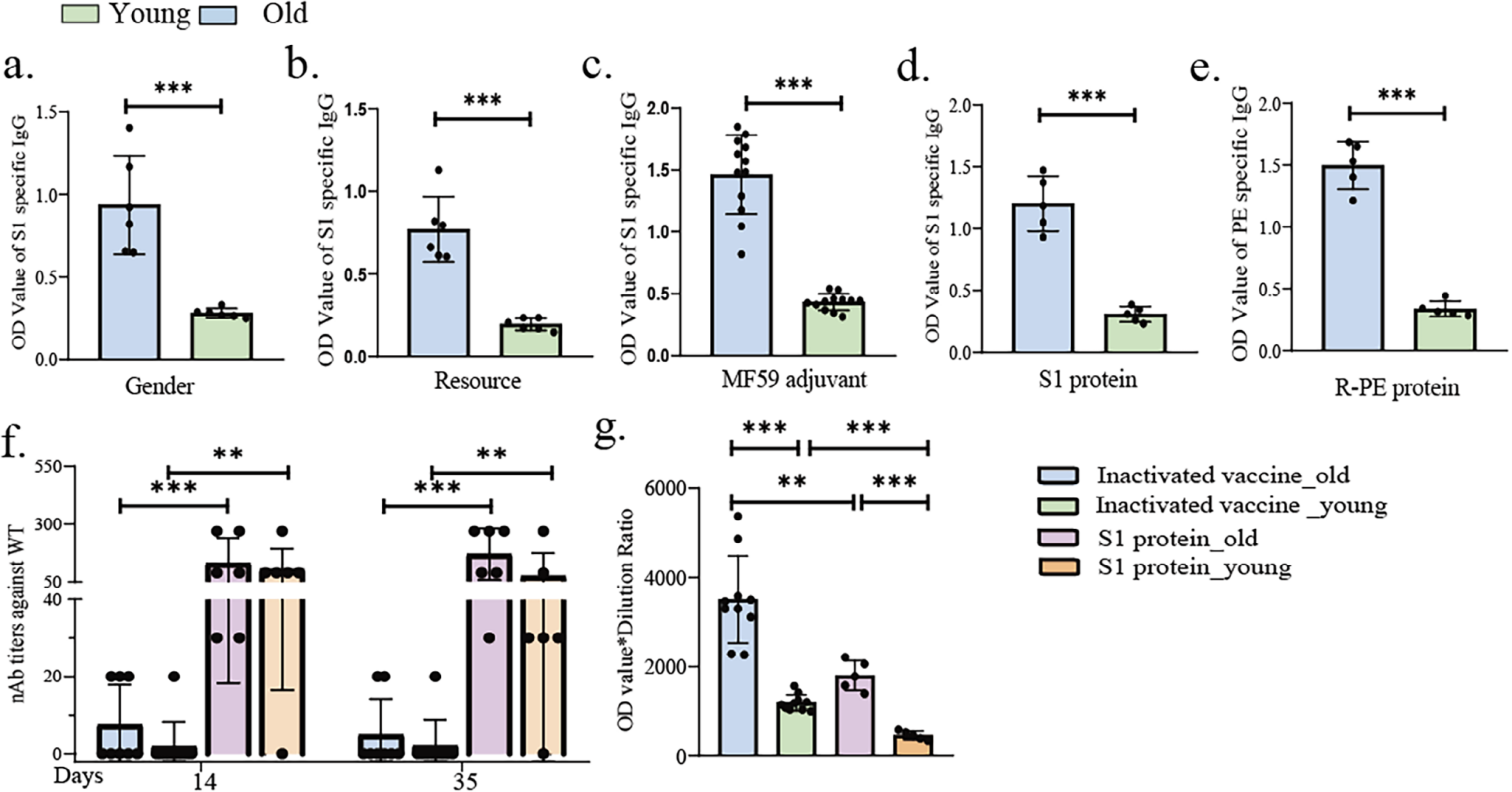
Enhanced antigen‐specific humoral responses in aged mice following immunogen challenge. a–c) S1‐specific antibody production at Day 14 post‐immunization in aged mice under modified immunization protocols: a) Varying mouse gender, b) Altering the mouse source, and c) Alternative adjuvant (MF59). d) S1‐specific antibody levels at Day 14 post‐immunization with S1 protein. e) PE‐specific antibody levels at Day 14 following PE protein immunization. f) Mice were immunized with the S1 protein/ SARS‐CoV‐2 inactivated vaccine on Day 0 and Day 21, and serum neutralizing antibody levels were evaluated on Day 14 and Day 35. g) A comparison of S1‐specific antibody levels in serum on Day 14 following immunization with S1 protein or the SARS‐CoV‐2 inactivated vaccine.

The paradigm of age‐related immunosenescence is widely accepted. However, we unexpectedly found that aged mice produced higher levels of antigen‐specific antibodies than young mice post‐immunization with the SARS‐CoV‐2 inactivated vaccine. To determine whether this phenomenon is generalizable, we investigated the different vaccination conditions, including not only mice with different genders and sources, but also vaccines with different adjuvants and antigens (**Figure** **2**a–e). For antigens, we used recombinant S1 protein from the SARS‐CoV‐2 virus or fluorescent protein, R‐Phycoerythrin (R‐PE), instead of the SARS‐CoV‐2 inactivated virus (Figure 2d,e). Consistently, aged mice exhibited elevated antigen‐specific antibody levels across all conditions, indicating that the enhanced antigen‐specific antibody responses in aged mice are a more general phenomenon. Interestingly, regardless of age, the inactivated virus vaccine induced relatively lower levels of NAbs than the recombinant S1 protein vaccines in both cohorts (Figure 2f), despite eliciting higher levels of S1‐specific IgG (Figure 2g). Additionally, similar to inactivated virus vaccine, age‐related differences in NAbs production were absent in recombinant S1 protein vaccinated mice (Figure 2f). Taken together, these findings suggested that aged mice exhibited universally enhanced antigen‐specific antibody responses (predominantly low‐affinity), but comparable NAbs levels to young mice, independent of gender, sources, adjuvant, or antigen.

### 2.3 Mechanistic Basis of Enhanced Humoral Immunity in Aged Mice

**Figure 3:**
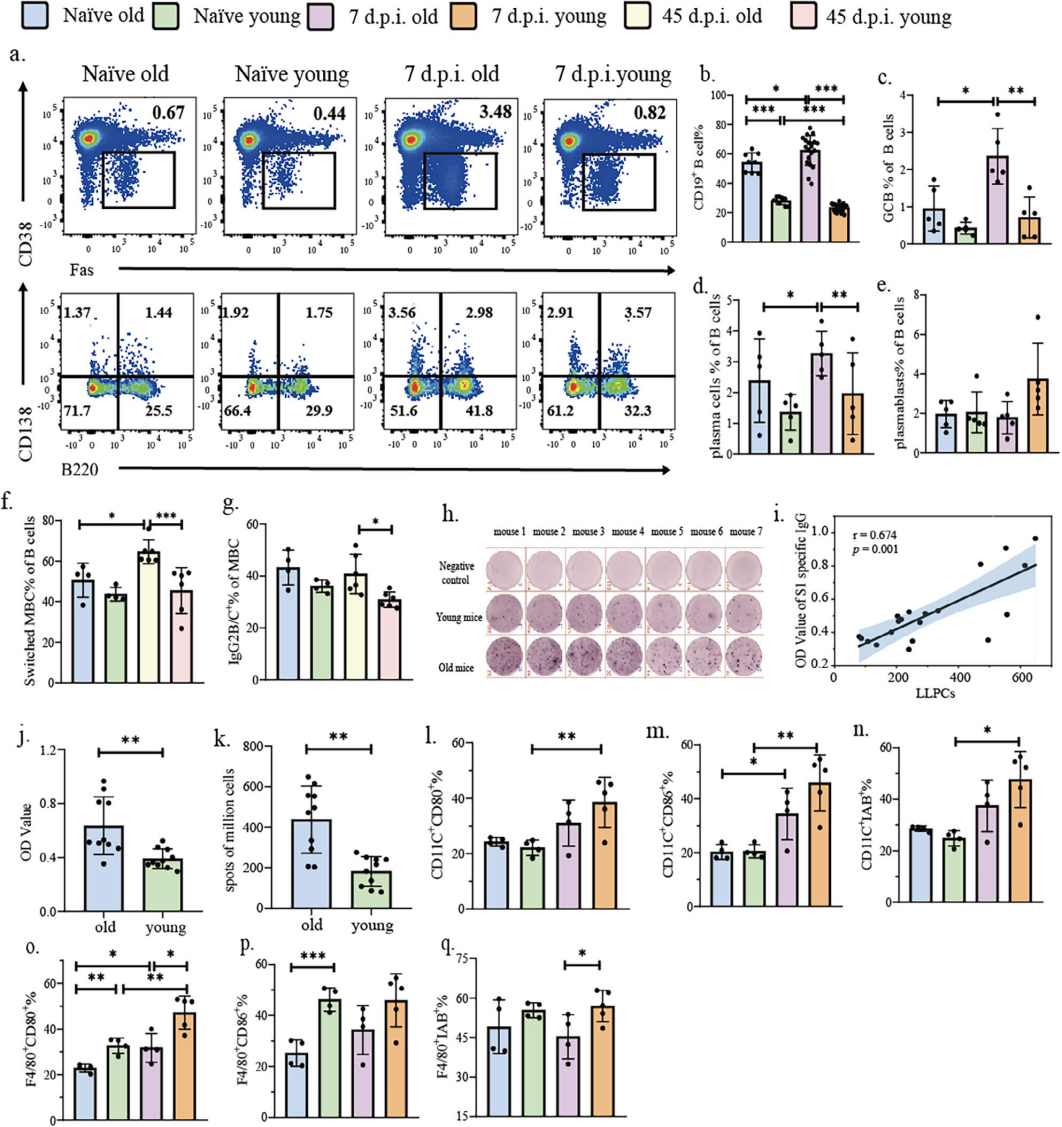
Humoral immune response and antigen‐presenting cell profiles in aged versus young mice following SARS‐CoV‐2 inactivated vaccination. a) Representative flow plots. b–e) The proportions of B cells, germinal center B cells (CD3^−^Dump^−^CD19^+^CD38^−^ FAS^+^), plasma cells(CD3^−^Dump^−^CD19^−^CD138^+^B220^−^), and plasmablasts(CD3^−^Dump^−^CD19^−^CD138^+^B220^+^) in the draining lymph nodes of aged and young mice at baseline and D7 after SARS‐CoV‐2 inactivated vaccination, respectively. f,g) The proportions of Class switched memory B cells (Dump^−^IgD^−^CD19^+^CD38^+^B220^+^) and IgG2b/c^+^ memory B cells (Dump^−^IgD^−^CD19^+^CD38^+^B220^+^IgG2b/c^+^) in the draining lymph nodes of aged and young mice at baseline and D49 after SARS‐CoV‐2 inactivated vaccination. Respectively. h) LLPCs representative spot plot. i) Correlation between the number of LLPC spots in bone marrow and the level of serum S1‐specific antibody 70 days after immunization with two doses of SARS‐CoV‐2 inactivated vaccine. j) Comparison of S1 antigen‐specific IgG levels in the serum of aged and young mice 70 days after two doses of SARS‐CoV‐2 inactivated vaccination. k) Comparison of LLPC number in bone marrow of aged and young mice 70 days after two doses of SARS‐CoV‐2 inactivated vaccination. l,m) The expression levels of CD80 and CD86 in DC cells in the draining lymph nodes of aged and young mice at baseline and D7 after SARS‐CoV‐2 inactivated vaccination, respectively. n) Expression of MHC‐II on DC cells in the draining lymph nodes of aged and young mice at baseline and D7 after SARS‐CoV‐2 inactivated vaccination. o,p) The expression levels of CD80 and CD86 in macrophages in the draining lymph nodes of aged and young mice at baseline and D7 after SARS‐CoV‐2 inactivated vaccination, respectively. q) Expression of MHC‐II on macrophages in the draining lymph nodes of aged and young mice at baseline and D7 after SARS‐CoV‐2 inactivated vaccination.

Upon exposure to immunogens, germinal centers (GCs) orchestrate humoral immunity by driving extensive proliferation of GC B cells (GCB), leading to their differentiation into plasma cells (PCs) and memory B cells (MBCs).^[^ ^22^ ^]^ PCs are responsible for secreting large quantities of high‐affinity antibodies,^[^ ^23^ ^]^ while MBCs enter the bloodstream for recycling and provide rapid immune responses upon re‐exposure to the same antigen.^[^ ^24^ ^]^ Consequently, GCBs, PCs, and MBCs are critical cellular subsets of humoral immunity. We found that aged mice displayed a higher proportion of B cells in draining lymph nodes (dLNs) at baseline than young mice and further amplified following vaccination (**Figure** **3**a,b). Although the proportions of GCBs, PCs, and MBCs were comparable at baseline in both groups, aged mice exhibited significantly elevated frequency of GCBs and PCs, but not plasmablasts, compared with young mice at day 7 post‐immunization (Figure 3c‐e, Figure S2a–c, Supporting Information). In addition, class‐switched MBCs were significantly increased at day 49 after immunization (Figure 3f). Among these subsets, the percentage of IgG2b/c^+^ MBCs, but not IgG1^+^MBCs, was significantly higher in aged mice than that of young mice (Figure 3g, Figure S2d, Supporting Information), consistent with the class‐switching profile of S1‐specific antibodies in serum (Figure 1). However, no differences in class‐switched MBCs were observed among the groups within the spleen (Figure S2e–g, Supporting Information). Long‐lived plasma cells (LLPCs) in bone marrow are critical to provide long‐lasting antibody responses.^[^ ^25^ ^]^ On day 70 after immunization with two doses of vaccine, when the acute immune response to the vaccine had subsided, the number of LLPCs, as well as the level of S1‐specific antibodies, in aged mice was significantly increased compared with young mice (Figure 3h–k). Moreover, the number of LLPCs in the bone marrow was positively correlated with the level of S1‐specific IgG in the serum (Figure 3i). These findings underscored the enhanced humoral immunity in aged mice following vaccination, characterized by increased GC responses, B cell differentiation, LLPCs persistence, as well as sustained production of S1‐specific antibodies.

### 2.4 Impaired Function of Antigen‐Presenting Cells in Aged Mice

The development of S1‐specific B cells requires myeloid‐T cell collaboration.^[^ ^26^ ^]^ To elucidate age‐related differences in humoral immunity induced by the SARS‐CoV‐2 inactivated vaccine, we analyzed the activation status and functionality of dendritic cells (DCs) and macrophages in dLNs by detecting the expression of CD80, CD86, and IAb on these cells. CD80 and CD86 are co‐stimulatory molecules,^[^ ^27^, ^28^ ^]^ and IAb represents antigen‐presentation ability of DCs and macrophages.^[^ ^29^ ^]^ We found that the expression levels of CD80, CD86, and IAb in DCs were comparable between aged and young mice before vaccination, but were dramatically upregulated post‐vaccination (Figure 3l–n, Figure S2h–j). However, the baseline expression levels of CD80 and CD86 in macrophages of aged mice were significantly reduced compared with young mice before vaccination, despite comparable absolute cell numbers between the two groups (Figure 3o,p, Figure S2k,l). CD80 expression in macrophages increased from the baseline in both groups post‐vaccination, yet remained significantly higher in young mice than in aged mice (Figure 3o). In contrast, the expression of CD86 in macrophages did not exhibit vaccination‐induced changes in either group (Figure 3p). Moreover, the expression level of IAb remained significantly higher in young mice compared with aged mice after vaccination (Figure 3q, Figure S2m, Supporting Information). Thus, consistent with much of the literature,^[^ ^30^, ^31^ ^]^ our study demonstrates that the function of mouse APCs is impaired to varying degrees during aging.

### 2.5 Age‐Related T Cell Characteristics and Post‐Vaccination Dynamics

**Figure 4:**
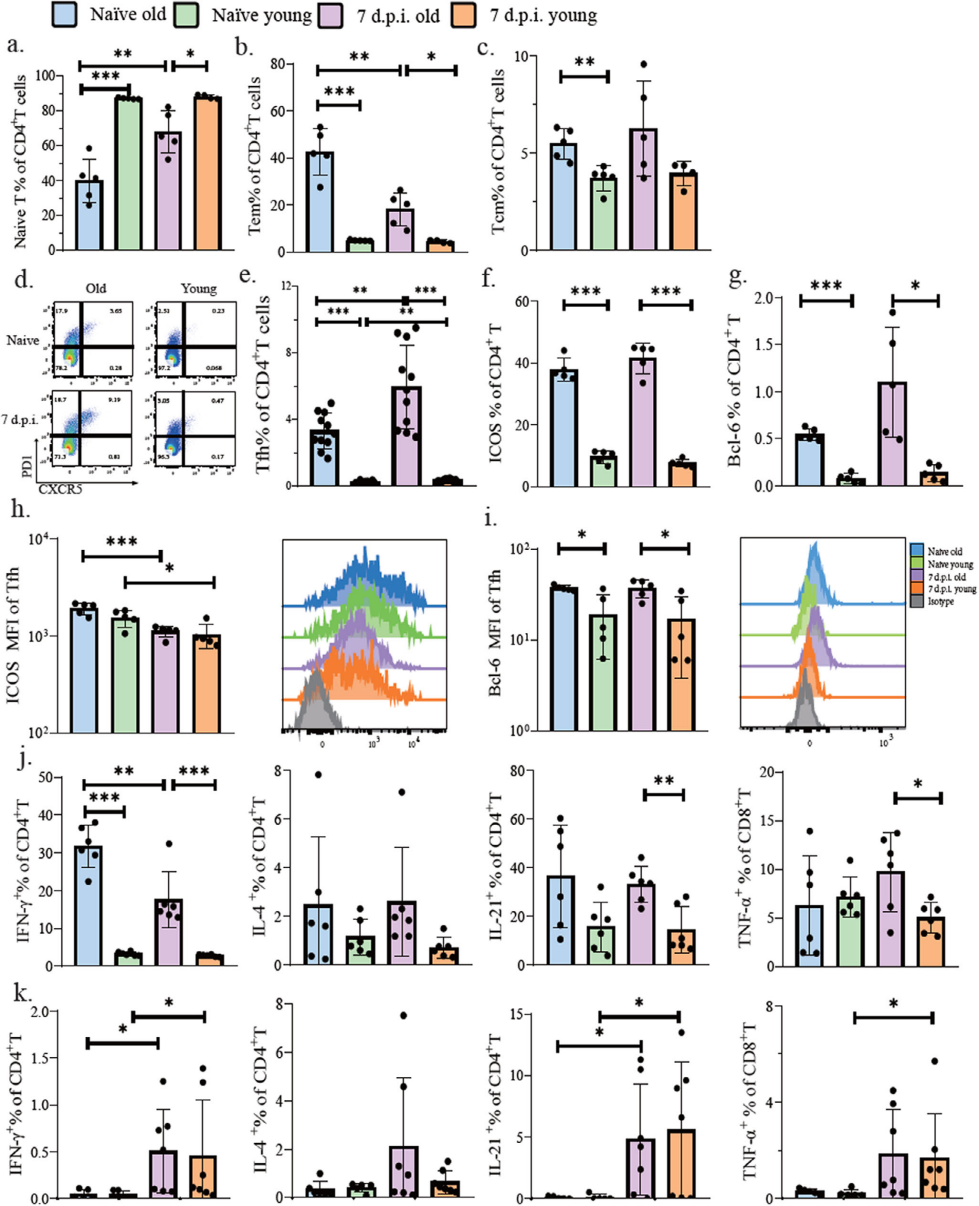
T cell characteristics of aged and young mice after SARS‐CoV‐2 inactivated vaccination. a–c) The proportions of naïve CD4^+^T cells and memory CD4^+^T cells in the draining lymph nodes of aged and young mice at baseline and D7 after SARS‐CoV‐2 inactivated vaccination, respectively. (Tcm, central memory T cell, CD3^+^Dump^−^Aqua^−^CD44^+^CD62L^+^; Tem, effector memory T cell, CD3^+^Dump^−^Aqua^−^CD44^+^CD62L^−^). d) Representative flow plots of Tfh cells(CD3^+^Dump^−^CD4^+^CXCR5^+^PD1^+^). e–g) The proportions of Tfh, ICOS(CD3^+^Dump^−^CD4^+^ICOS^+^), and Bcl‐6 (CD3^+^Dump^−^CD4^+^Bcl‐6^+^)in the draining lymph nodes of aged and young mice at baseline and D7 after SARS‐CoV‐2 inactivated vaccination, respectively. h) ICOS MFI of Tfh. i. Bcl‐6 MFI of Tfh. MFI, Mean fluorescence intensity. j) The proportion of CD4^+^T cells secreting IFNγ, IL‐4, IL‐21, and CD8^+^T cells secreting TNF‐α in the draining lymph nodes of aged and young mice at baseline and D7 after SARS‐CoV‐2 inactivated vaccination were measured after 6 hours of stimulation with PMA and ionomycin. k) The proportion of antigen specific CD4^+^T cells secreting IFNγ, IL‐4, IL‐21, and antigen specific CD8^+^T cells secreting TNF‐α in the draining lymph nodes of aged and young mice at baseline and D7 after SARS‐CoV‐2 inactivated vaccination were measured after 6 hours of stimulation with SARS‐CoV‐2 S1 peptide library.

We next interrogated age‐related lymphocyte profiling in dLNs. B cells constituted the predominant population in aged mice, contrasting with the T cell dominance observed in young counterparts (Figure S2n, Supporting Information). Particularly, aged mice exhibited an elevated proportion of memory T cells and a corresponding reduction in naïve T cell populations compared to young controls (**Figure** **4**a–c, Figure S2o–w, Supporting Information). Furthermore, we observed CD8^+^T cell accumulation and an inverted CD4/CD8 ratio in aged mice (Figure S2x,y, Supporting Information). Given the pivotal role of follicular helper T (Tfh) cells in humoral immunity,^[^ ^32^ ^]^ we characterized these cells via PD‐1 and CXCR5 co‐expression to assess age‐related changes (Figure 4d). Surprisingly, aged mice displayed a tenfold higher frequency of Tfh cells compared to young mice, independent of vaccination status (Figure 4e). Consistent with this finding, aged mice showed significantly elevated expression levels of Bcl‐6 (the master transcriptional regulator of Tfh differentiation^[^ ^33^ ^]^) and ICOS (a key co‐stimulatory marker^[^ ^34^ ^]^) (Figure 4f–i). CD4^+^T cells could also polarize into different T helper subsets, secreting key cytokines to affect humoral responses.^[^ ^35^ ^]^ We examined the IFNγ, IL‐4, and IL‐21 expression in CD4^+^T cells. Except for IL‐4, the frequency of IFNγ, IL‐21‐producing CD4^+^T cells were higher post‐vaccination in aged mice compared with young mice (Figure 4j). Particularly, aged mice demonstrated a tenfold higher (31.68 ± 5.62% versus 3.31 ± 0.48%) frequency of IFNγ^+^ CD4^+^ T cells than that of young mice before vaccination (Figure 4j). However, absolute cell counts revealed significant intergroup differences only in IFNγ^+^CD4^+^T cells (Figure S3a–e, Supporting Information). We next asked if these cytokine‐producing CD4^+^T cells are antigen‐specific. By stimulating with the S1 protein peptide pool, antigens of SARS‐CoV‐2 specific CD4^+^T and CD8^+^T cells were barely detectable pre‐vaccination, but expanded comparably in both groups post‐vaccination (Figure 4k, Figure S3f–j, Supporting Information). These data suggested that Th1 cells accumulated in aged mice represented bystander T cells rather than vaccine‐induced antigen‐specific cells, and antigen‐specific CD4^+^ T cell induction was independent of age.

### 2.6 Single‐Cell Sequencing Reveals Transcriptomic Characteristics of CD4^+^T and B Cells in Aged Mice

**Figure 5:**
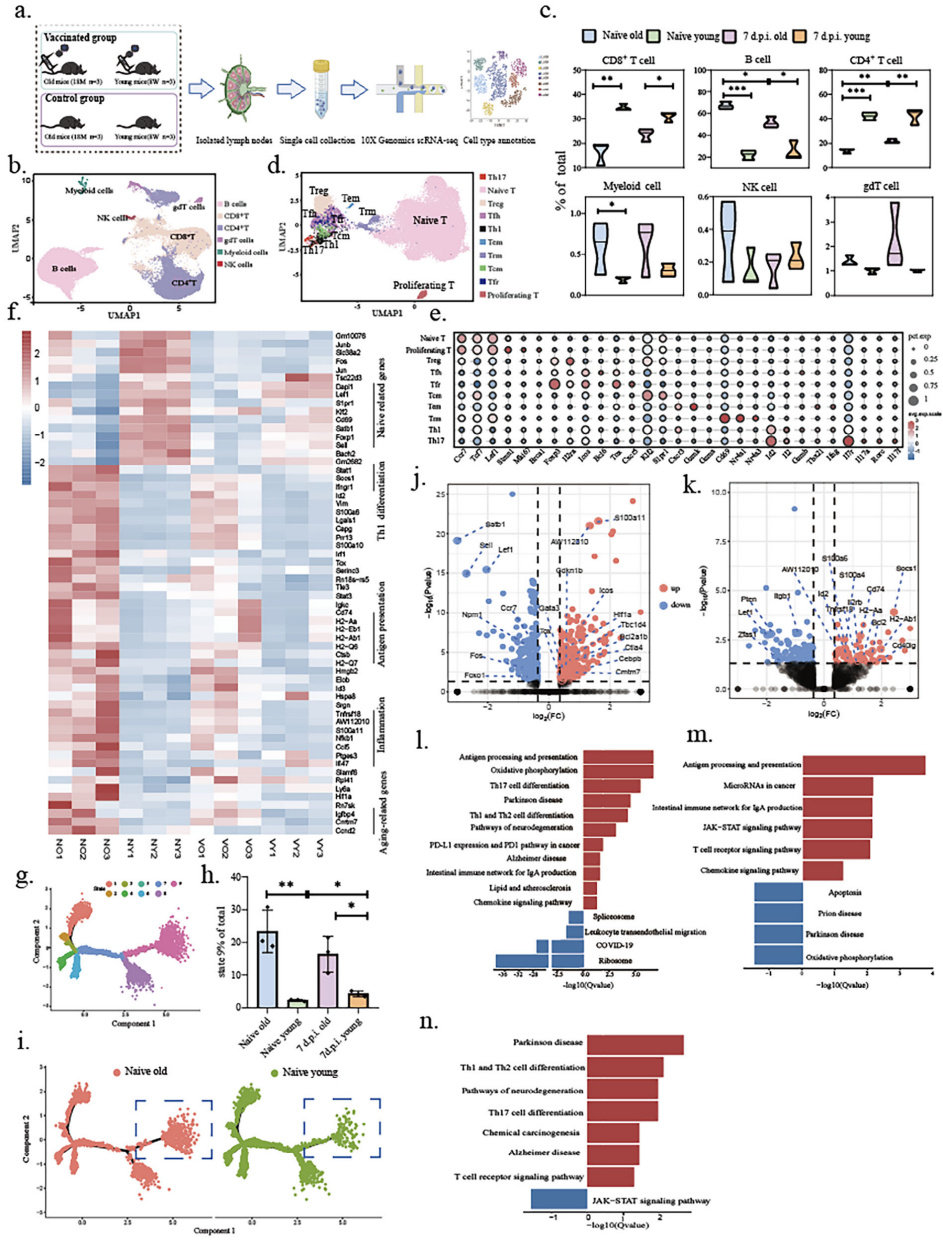
Single cell sequencing reveals transcriptomic characteristics of CD4^+^T cells in aged mice. a) Schematic of the process. b) UMAP analysis displays the clustering of immune cells in the draining lymph nodes. c) Statistical map of immune cell clusters in draining lymph nodes. d) Identification of CD4^+^ T cell subpopulations. e) marker gene bubble map of CD4^+^T cell subgroups. Horizontal coordinate is the gene name, and vertical coordinate is the subpopulation information of cells. The size of bubbles represents the proportion of cells expressing the corresponding gene, and the color depth represents the average gene expression. f) Differential gene heatmap of CD4^+^ T cells in aged and young mice at baseline and D14 after vaccine immunization. g) CD4^+^T cell differentiation trajectory diagram, different colors represent cells with different differentiation states. h) Statistical analysis of the proportion of state 9. i) State 9 distribution of CD4^+^T cells at baseline in aged and young mice. j,k) Volcano map of differential genes in tfh(j) and th1 cells(k) at baseline. The red dot represents the up‐regulated differential genes in aged mice, and the blue dot represents the down‐regulated differential genes in aged mice. Gray dots indicate genes that do not differ. l,m) KEGG enrichment analysis showed the differential enrichment pathway of Tfh cells at baseline and D14 after SARS‐CoV‐2 inactivated vaccination. n) KEGG enrichment analysis showed the differential enrichment pathway of Th1 cells at baseline. Red color represents significant upregulation in aged mice. Blue represents significant up‐regulation in young mice.

Our previous results suggested the age‐related enhancement function of B cells, impairment of myeloid cells' activity, and Th1 responses polarization. To elucidate cellular mechanisms underlying age‐enhanced humoral responses, we performed single‐cell RNA sequencing on dLN cells from young and aged C57BL/6 mice pre‐ and post‐vaccination (**Figure** **5**a). After rigorous quality control, 63 843 high‐quality cellular profiles were analyzed, and six major cell subsets were identified by using established immune‐related marker genes (Figure 5b). The observed age‐dependent shift toward B cell predominance and T cell reduction confirmed our flow cytometry findings (Figure 5c). A Sub‐clustering of B cells identified six subtypes based on the upregulated gene characteristics in each cluster (Figure S6a–d, Supporting Information). Aged B cells exhibited marked transcriptional dysregulation, with 17 upregulated genes, including tumor‐related genes (*Zfas1, Dnaja1*),^[^ ^36^, ^37^ ^]^ genes associated with protein homeostasis (*Crip1, Psmb9*),^[^ ^38^ ^]^ and inflammation‐related genes (*Ifi30, AW112010*).^[^ ^39^, ^40^ ^]^ Conversely, inflammation‐suppressive genes (*Tsc22d3*),^[^ ^41^ ^]^ cell cycle regulation (*Jun, Btg2*)^[^ ^42^ ^]^ and immune cell regulation and activation (*Junb, Cd69, Zfp36l1, Cd24a, Ccr7*)^[^ ^43^, ^44^, ^45^ ^]^ were suppressed (Figure S6e,f, Supporting Information). KEGG pathway enrichment analysis of these differential genes revealed their primary association with oxidative phosphorylation, Th1/ Th2 cell differentiation, PD‐L1 expression and PD1‐related pathways, Th17 cell differentiation, Toll‐like receptor signaling pathways, and cellular senescence‐related pathways (Figure S6g, Supporting Information). These findings collectively demonstrate that aged B cells acquired multiple senescence‐associated phenotypes, including cell cycle arrest, heightened inflammatory responses, and impaired regulatory capacity.

A Sub‐clustering of T cells identified ten subtypes (Figure 5d,e). Compared to young mice, a total of 162 upregulated and 55 downregulated genes were identified in the baseline CD4^+^ T cells of aged mice. Upregulated genes included antigen presentation (*Cd74, H2‐Aa, H2‐Q6, H2‐Eb1, H2‐Ab1, H2‐Q7, Ctsb*),^[^ ^46^, ^47^ ^]^ inflammation‐related genes (*AW112010, Ccl5, S100a11, Ptges3, Ifi47, Nfkb1, Ifngr1*),^[^ ^48^, ^49^ ^]^ T cell activation‐related genes (*Tnfrsf18, Slamf6*),^[^ ^50^ ^]^ aging‐related phenotypic markers (*Igfbp4, Ifngr1, Ccnd2, Cmtm7*),^[^ ^49^, ^51^ ^]^ effector‐related genes (*S100a6, Id2, Ly6a*),^[^ ^50^ ^]^ and Th1 differentiation‐related genes (*Socs1, Stat1, Ifngr1*).^[^ ^52^, ^53^ ^]^ In contrast, downregulated genes associated with naive (*Lef1, Satb1, Dapl1, Klf2, Foxp1, S1pr1, Sell*)^[^ ^48^, ^50^, ^54^ ^]^ and immune cell regulation (*Junb, Cd69*)^[^ ^55^, ^56^ ^]^ (Figure 5f, Figure S6h, Supporting Information). Cell trajectory analysis was performed using the Monocle tool (Figure S6i, Supporting Information), which classified cells into different states based on their differentiation status (Figure 5g). We found that an age‐related preference for differentiation into state 9 (Figure 5h,i, Figure S6j, Supporting Information), which predominantly contained Tfh and Th1 cells (Figure S6k, Supporting Information). Thus, naive CD4^+^ T cells in aged mice are more inclined to differentiate into Tfh and Th1 cells.

Single‐cell transcriptional profiling of Tfh and Th1 cell subsets revealed more pronounced age‐associated differential gene expression (DEGs) in pre‐vaccination compared to post‐vaccination. Particularly, upregulated DEGs in pre‐vaccination related to inflammation, antigen presentation, effector functions, cell cycle regulation, and aging‐related phenotypes (Figure 5j,k). Aged Tfh cells exhibited increased expression of genes (*IL2rb, Ctla4, Id2, and Il7r*) that with negative regulation of Tfh cell differentiation (Figure 5j). Specifically, *IL‐2rb* may directly influence the expression of PD‐1,^[^ ^57^ ^]^ the DNA‐binding protein *Id2* inhibits the expression of CXCR5,^[^ ^58^ ^]^ *Ctla4* regulates humoral immunity by downregulating co‐stimulatory molecules B7‐1 and B7‐2,^[^ ^59^ ^]^ and *Il7r* antagonizes the function of Bcl6 via the IL7R/STAT5 axis.^[^ ^60^ ^]^ Additionally, Aged Tfh cells downregulated genes related to GC response, such as *Npm1*,^[^ ^61^ ^]^ *Foxo1*, and *Lef1*.^[^ ^34^, ^62^ ^]^ KEGG pathway enrichment analysis of these differential genes revealed upregulation of pathways related to antigen processing and presentation, Th1 and Th2 differentiation, and oxidative phosphorylation, while pathways associated with COVID‐19 were downregulated (Figure 5l). After vaccination, aging Tfh cells exhibit upregulation of pathways such as antigen processing and presentation, T cell receptor signaling, JAK‐STAT signaling, and chemokine signaling, while apoptosis‐related pathways are inhibited (Figure 5m). In parallel, aged Th1 cells showed upregulation of T cell survival and activation (*Bcl2, Tnfrsf18*),^[^ ^63^ ^]^ alongside elevated expression of Cd40lg encoding CD40 ligand (CD40L) (Figure 5k). This surface‐expressed molecule on T helper cells engages CD40 on B cells to drive their proliferation, differentiation, and immunoglobulin class‐switching.^[^ ^64^ ^]^ Upregulated DEGs in aged Th1 cells were enriched in Th1/Th2/Th17 differentiation and T cell receptor signaling, and downregulated DEGs enriched JAK‐STAT signaling (Figure 5n). However, post‐vaccination analysis showed marked attenuation of age‐related transcriptional differences. Th1 cells exhibited complete loss of pathway enrichment post‐vaccination. These findings collectively demonstrate that aging predominantly impacts T cell functionality through the accumulation of a pre‐activated Th cell compartment in unvaccinated aged mice, whereas vaccination‐induced immune activation mitigating age‐related transcriptional disparities.

### 2.7 Aged CD4^+^T Cells Promote S1‐Specific Antibody Production Following SARS‐CoV‐2 Inactivated Vaccination

**Figure 6:**
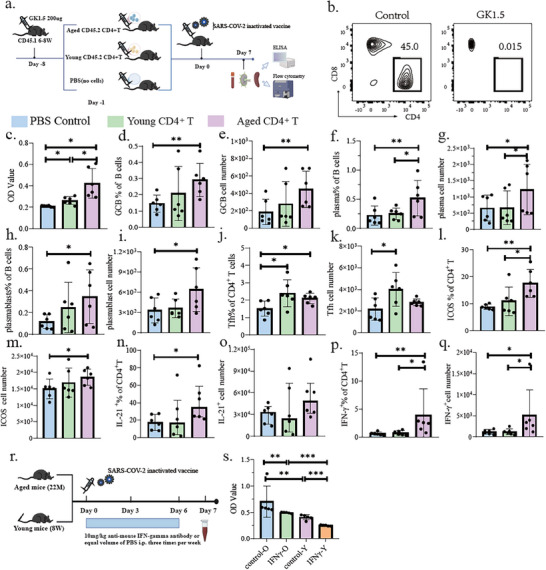
Aged CD4^+^ T cells promote S1‐specific antibody production after SARS‐CoV‐2 inactivated vaccination. a) Experimental workflow for CD4^+^ T cell adoptive transfer. b) Proportions of CD4^+^ and CD8^+^ T cells in dLNs 7 days post 200 µg GK1.5 intraperitoneal injection. c) Comparison of S1‐specific antibody levels in the serum of CD45.1 mice 7 days after SARS‐CoV‐2 inactivated vaccination, following adoptive transfer of CD4^+^ T cells from different sources. d–q) Comparisons of GCB, PCs, plasmablasts, Tfh cells, ICOS, IL‐21‐secreting CD4^+^ T cells, Th1 cells, and their absolute cell counts in draining lymph nodes 7 days post SARS‐CoV‐2 inactivated vaccination in CD45.1 mice, following adoptive transfer of CD4^+^ T cells from different sources. r) Diagram of the IFNγ neutralization experiment. s) Comparison of S1‐specific antibody levels in aged and young mice following SARS‐CoV‐2 inactivated vaccination, after IFNγ depletion.

To delineate the function of CD4^+^T cells in promoting age‐related humoral responses, we established a CD4^+^ T cell adoptive transfer model (**Figure** **6**a) in which CD4^+^ T cells were depleted in CD45.1 C57BL/6 mice (6–8 weeks) by intraperitoneally injecting anti‐CD4 antibody (GK1.5), followed by adoptively transfer of enriched CD4^+^ T cells from CD45.2 aged or young donor C57BL/6 mice, with PBS as a control (Figure 6b). Blood/tissue samples were collected for immunological assays at day 7 post‐vaccination. Notably, both young and aged donor CD4^+^ T cells restored S1‐specific antibody production compared to PBS controls (Figure 6c), yet aged CD4^+^ T cells elicited markedly elevated antibody titers compared to young counterparts (Figure 6c). This enhancement correlated with expanded germinal center B cells and heightened plasma cell and plasmablasts, suggesting amplified germinal center activity in aged CD4^+^ T cell recipients (Figure 6d–i). Although Tfh cells were elevated in both aged and young CD4^+^ T cell transplantation groups compared to PBS controls, the proportion and absolute numbers of Tfh cells, as well as IL‐21‐producing CD4^+^T cells, were comparable between aged and young CD4^+^ T cell transplantation groups (Figure 6j–o). On the other hand, aged CD4^+^ T cell recipients displayed distinct Th1 polarization characterized by elevated proportions and absolute counts of IFNγ^+^ CD4^+^T cells (Figure 6p,q). Although the subcutaneous administration of the SARS‐CoV‐2 inactivated vaccine means the dLNs are the primary site of the immune response, the spleen, as a major peripheral immune organ, plays a crucial role in host defense and overall immune regulation. Therefore, we also assessed changes in the splenic immune cell landscape. In contrast to the dLNs, the composition of most immune cell populations in the spleen did not differ significantly among the three groups (Figure S7a–l, Supporting Information). Nevertheless, the proportion of IFNγ^+^ CD4^+^T cells was significantly elevated in the spleens of aged CD4^+^ T cell transplantation groups compared to the other two groups(Figure S7m,n), implicating Th1 skewing as a conserved feature of aged CD4^+^ T cell responses. Furthermore, we employed an IFN‐γ neutralization model (Figure 6r). The results demonstrated that neutralization of IFN‐γ in mice led to a significant reduction in S1‐specific antibody levels in the sera of both aged and young mice, although the difference between the two groups persisted (Figure 6s). This underscores the importance of IFN‐γ in antibody production post‐vaccination. Based on the abundance of immune cells in lymph nodes and their respective proportions of IFN‐γ production, we ruled out B cells and myeloid cells as the primary sources. Interestingly, among IFN‐γ‐producing T cells, CD4^+^ and CD8^+^ T cells were present in comparable proportions (Figure S7o, Supporting Information).

### 2.8 Baseline Th1 Cell Frequency Predict Antibody Magnitude Following SARS‐CoV‐2 Inactivated Vaccination

**Figure 7:**
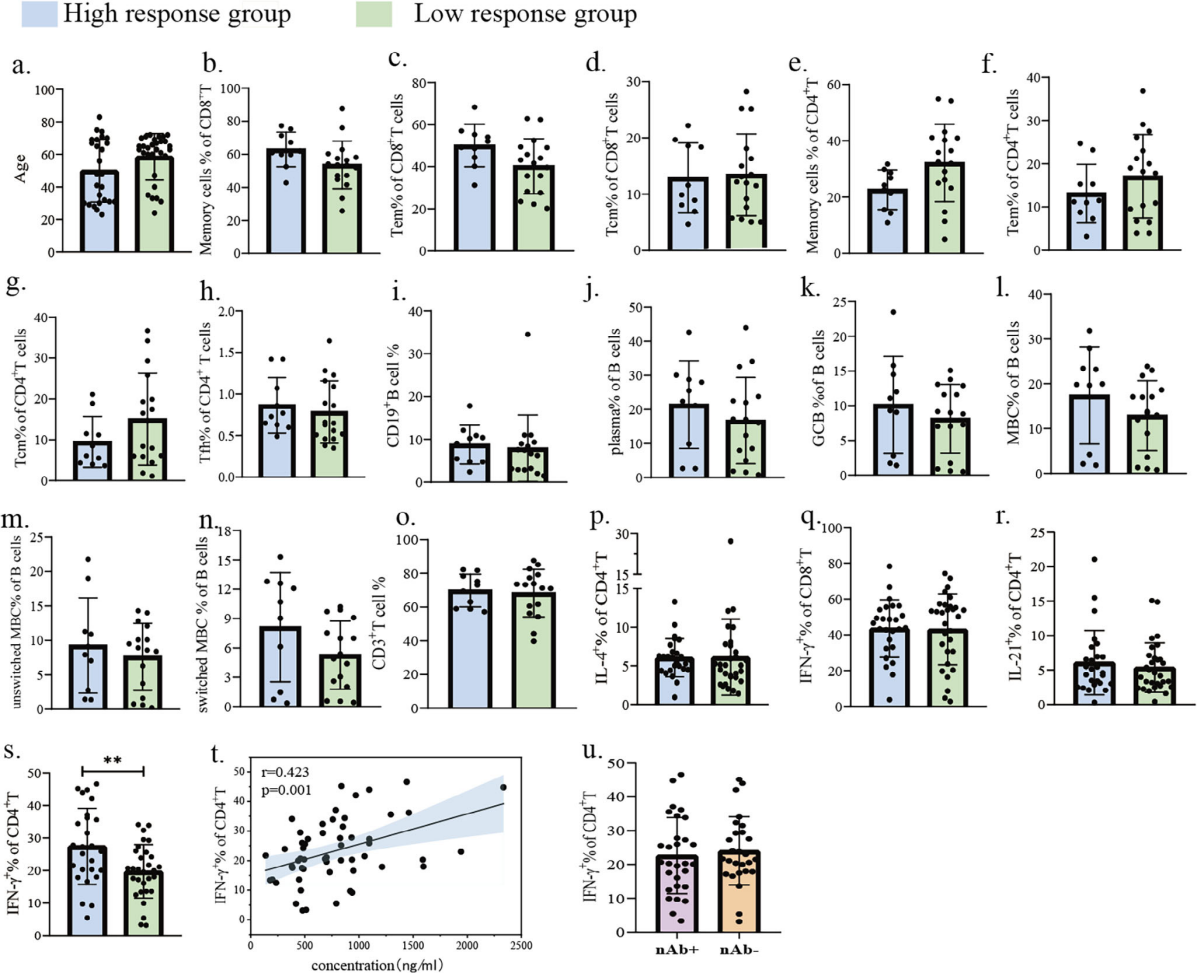
Comparison of baseline immune cells in high and low antibody responders post‐ SARS‐CoV‐2 inactivated vaccination. a) Comparison of age between the high and low antibody response groups. b–r) Comparison of baseline immune cell subsets between high and low antibody response groups, including CD8^+^ memory cells, CD8^+^ Tem, CD8^+^ Tcm, CD4^+^ memory cells, CD4^+^ Tem, CD4^+^ Tcm, Tfh cells, B cells, plasma cells, GCB cells, MBCs, unswitched MBCs, switched MBCs, T cells, CD4^+^ IL‐4^+^ cells, CD8^+^ IFNγ^+^ cells, and CD4^+^ IL‐21^+^ cells. s) Comparison of Th1 levels at baseline between the high and low antibody response groups. t) Correlation between baseline Th1 cells and S1‐specific antibody levels post‐vaccination. u) Comparison of Th1 levels at baseline between neutralizing antibody‐positive and neutralizing antibody‐negative groups post‐SARS‐CoV‐2 inactivated vaccination.

To investigate whether murine phenotypes translate to humans, we conducted a prospective cohort study of healthy volunteers stratified by age (≥65 versus 18–64 years) receiving single‐dose SARS‐CoV‐2 inactivated vaccine (Figure S8, Table S1, Supporting Information). Comprehensive pre‐vaccination immunophenotyping revealed comparable baseline proportions of major lymphocyte subsets—including T cell subsets, B cell compartments, and cytokine‐producing populations—between age groups (Figure S8, Supporting Information). Furthermore, no differences in RBD‐specific IgG production were observed between the elderly and young groups at post‐vaccination Day 28(Table S1, Supporting Information). Even when employing a more stringent age classification, with the “elderly” group (≥65 years) and the “young” group (between 18 and 35 years), our conclusion remains consistent (Figure S8s,t, Supporting Information). While advanced age has been associated with diminished vaccine responses in prior studies,^[^ ^17^ ^]^ our data suggested individual heterogeneity and environmental influences may override chronological age as predictors of humoral immunity, underscoring the need for mechanistically grounded biomarkers. Reclassifying participants by post‐vaccination RBD‐specific antibody levels (high versus low responders) revealed no demographic or cellular composition differences in conventional immune subsets (**Figure** **7**a–r). Strikingly, high responders exhibited elevated pre‐existing Th1 cell frequencies compared to low responders (Figure 7s). This association was quantitatively reinforced by a significant positive correlation between baseline Th1 proportions and RBD‐specific antibody titers (r = 0.423, p = 0.001; Figure 7t), contrasting with the lack of correlation observed for IFNγ^+^ CD8^+^ T cells (Figure S9b, Supporting Information).or other measured subsets (Figure S9, Supporting Information). Notably, Th1 frequency did not distinguish neutralizing antibody serostatus (Figure 7u), suggesting its influence operates through quantitative modulation rather than seroconversion threshold effects. We subsequently performed a multivariable linear regression analysis adjusted for age, sex, and hypertension status. After adjusting for these potential confounders, the baseline Th1 level remained a significant independent predictor of antibody titer (p < 0.001; Table S2, Supporting Information).

## 3 Discussion

COVID‐19 represents a significant global public health priority.^[^ ^65^ ^]^ While substantial research attention has focused on identifying factors contributing to inconsistent vaccine responses, host‐related determinants frequently receive inadequate consideration. Advanced age constitutes a well‐established factor influencing vaccine immunogenicity,^[^ ^66^, ^67^ ^]^ however, investigations evaluating vaccination outcomes in elderly populations present considerable challenges. These difficulties arise not only from the pronounced heterogeneity in immune responses observed with advancing age but also from the high prevalence of underlying comorbidities within aging cohorts.^[^ ^68^ ^]^ Although suboptimal vaccine‐induced immunity in elderly adults is often attributed to immunosenescence and associated alterations in immune composition,^[^ ^69^ ^]^ our data reveal that the concentrations of antigen‐specific antibodies elicited post‐vaccination demonstrate no statistically significant difference between elderly and younger populations. Furthermore, substantial inter‐individual variation exists in the compositional landscape of immune cells among human subjects, which cannot be fully attributed to chronological age alone. Consequently, the identification of novel, objective predictive biomarkers for vaccine immunogenicity assumes critical importance in refining immunization strategies.

Th1 cells orchestrate critical effector functions by enhancing phagocyte‐mediated defense mechanisms against intracellular bacterial and viral pathogens.^[^ ^70^ ^]^ Through secretion of IFN‐γ, these lymphocytes activate key transcription factors (STAT1, AP‐1, and NF‐κB) within B cells, thereby driving B cell differentiation and promoting immunoglobulin class switching to IgG2a/2c or IgG2b isotypes.^[^ ^71^ ^]^ Our investigation demonstrates that baseline Th1 cell frequency strongly predicts protective efficacy following immunization with SARS‐CoV‐2 inactivated vaccines. Notably, aged mice exhibiting polarized CD4⁺ T cell profiles with elevated Th1 frequencies generated significantly higher titers of SARS‐CoV‐2‐specific antibodies than young mice upon vaccination, predominantly of the IgG2c subclass, and mounted immune responses skewed toward a Th1 phenotype. The increased Th1 cells were bystander CD4^+^ T cells, as no significant differences were observed between aged and young mice regardless of vaccination (Figure 4k). Functional validation through IFN‐γ depletion in vivo resulted in markedly reduced production of vaccine‐elicited SARS‐CoV‐2‐specific antibodies (Figure 6s), confirming the indispensable role of Th1 cells. These findings provide novel mechanistic insights into vaccine immunogenicity and bear significant implications for the rational design of next‐generation COVID‐19 vaccines with enhanced protective capacity. Furthermore, our findings hold important implications for addressing viral evolution and guiding future vaccination strategies. Our research underscores the critical importance of the “individual”s baseline immune status,' providing a theoretical foundation for personalized immunization strategies. Assessing an individual's pre‐vaccination Th1 cell immunity helps identify potential “low‐responders,” who may benefit from tailored vaccination approaches. These could include adjusted dosage, additional booster shots, the use of different vaccine platforms, or customized booster regimens to achieve optimal protection. Moreover, the polyclonal antibodies generated with Th1 cells may confer protection through non‐neutralizing mechanisms, such as Fc‐mediated effector functions, which could become increasingly important against variants of concern. The measurement of Th1 cells (e.g., via IFN‐γ detection by flow cytometry) is a standardized clinical procedure. This immune profiling could potentially be integrated into large‐scale vaccination campaigns, particularly for high‐risk groups like the elderly or immunocompromised individuals. To realize this potential, future large‐scale prospective clinical studies are needed to validate a cutoff value for baseline Th1 cell frequency that predicts suboptimal vaccine response with high sensitivity and specificity, and integrating baseline Th1 cell measurement with other parameters (e.g., serological markers or metabolomic profiles) could help build a more robust predictive model. Furthermore, our animal models revealed that elevated antigen‐specific antibody responses in Th1‐enriched aged mice are not restricted to the SARS‐CoV‐2 inactivated vaccine platform, indicating broader applicability across diverse vaccine formulations. We therefore propose that Th1 cells may be a biomarker to predict the level of antibody production during immunization. Our findings closely align with the research by Lisa E. Wagar et al.,^[^ ^72^ ^]^ which demonstrated in an influenza vaccination model that baseline Th1 cells, rather than age, were significantly correlated with antibody responses. This observation is entirely consistent with our results from the SARS‐CoV‐2 inactivated vaccine. The consistency across different vaccine platforms (inactivated vaccines versus multivalent influenza vaccines) and distinct pathogens (coronavirus versus influenza virus) strongly suggests that T‐cell background immunity plays a previously underestimated central role in shaping subsequent antibody responses. This challenges the traditional paradigm of relying solely on age to predict vaccine efficacy and highlights the potential of personalized immunization strategies based on immune biomarkers. However, future studies are warranted to validate this concept across a broader range of vaccine platforms, such as mRNA and adenovirus‐vectored vaccines.

Comprehensive profiling of antigen‐specific T cells revealed minimal detectable antigen‐reactive populations at baseline, with the majority exhibiting bystander phenotypes. We propose that SARS‐CoV‐2 inactivated vaccines may activate Th1 cells through bystander activation mechanisms, consequently augmenting B cell‐mediated production of antigen‐specific antibodies. Our single‐cell transcriptomic analysis revealed a significant upregulation of CD40 ligand (Cd40lg) expression in aged Th1 cells compared to young mice. CD40L is an indispensable costimulatory molecule for T cell‐dependent B cell activation, antibody class switching, and germinal center formation.^[^ ^73^, ^74^ ^]^ Its elevated expression suggests that aged Th1 cells possess enhanced helper potential, enabling them to more effectively promote B cell clonal expansion, antibody class switching, and high‐level antibody production. Further pathway enrichment analysis demonstrated that, compared to young mice, aged Th1 cells exhibit an activated state under coordinated multi‐pathway regulation: pathways involved in antigen processing and presentation, T cell receptor signaling, JAK‐STAT signaling, and chemokine signaling were significantly upregulated, whereas apoptosis‐related pathways were markedly suppressed. This overall expression profile collectively indicates that aged Th1 cells reside in a “hyper‐responsive” and “long‐lived” functional state. Specifically, upregulation of chemokine signaling pathways enhances the chemotactic and positioning capacity of these cells, allowing more efficient migration to B cell follicles and facilitating essential T–B cell contact. Enhanced antigen presentation and TCR signaling pathways increase their sensitivity to antigenic stimulation and improve early activation efficiency. Meanwhile, downregulation of apoptotic pathways prolongs cell survival, thereby maintaining a more extended window for providing help. Thus, despite the overall context of immunosenescence, this compensatory functional enhancement of Th1 cells during aging—characterized by increased expression of key helper molecules, optimized migration and activation capacity, and prolonged survival—collectively overcomes other age‐related immune deficiencies. These mechanisms jointly contribute to the stronger humoral immune response and elevated antibody levels observed in aged mice.

Following SARS‐CoV‐2 inactivated vaccination, aged mice generated higher levels of S1‐specific antibodies than young mice, yet showed only marginally increased or comparable nAb titers. Similarly, in human cohorts, baseline Th1 cells correlated with RBD‐specific antibodies but not with nAbs. Thus, baseline Th1 cells serve as an excellent predictor of response magnitude but not of response quality, strongly suggesting that total antibody titers and nAb titers are governed by partially overlapping yet distinct immunological pathways. Furthermore, total IgG serves as the foundation and source of functional nAbs. Changes in total IgG titers are a prerequisite for the observed differences in nAb production. In our study, the differences in total antibody titers induced by inactivated vaccination were moderate in magnitude. Thus, within this context of a relatively limited immune response amplitude, the absence of significant differences in nAbs is biologically reasonable and logical. Baseline Th1 cell frequency is significantly correlated with vaccine‐induced binding antibody levels, but not with functional nAb titers. This phenomenon clearly indicates a dissociation between “quantity” and “quality” in Th1‐mediated promotion of humoral immunity. We propose that baseline Th1 cells primarily provide the “driving force” for expanding the B cell response, whereas the efficient production of high‐affinity nAbs relies more heavily on subsequent refined processing within the germinal centers. Therefore, although Th1 cells can aid B cells in antibody production, the protective efficacy of these antibodies requires further verification. Inactivated vaccines employ physicochemical pathogen inactivation to abolish replicative capacity while retaining immunogenicity.^[^ ^75^ ^]^ This platform's inherent safety profile—stemming from non‐replicating antigens that preclude virulence reversion or persistent infection—confers broad applicability across pediatric, geriatric, and comorbid populations.^[^ ^76^ ^]^ However, substantial evidence indicates suboptimal induction of nAbs and T cell immunity by this platform.^[^ ^77^, ^78^ ^]^ The absence of significant differences in post‐vaccination nAb titers or antigen‐specific T cell responses between high versus low baseline Th1 subgroups may reflect inherent immunogenicity limitations of inactivated vaccines. Notably, comparative immunization with S1 recombinant protein vaccines at equivalent doses demonstrated that although S1‐specific antibody titers were lower than those induced by inactivated vaccines (Figure 2g), nAb levels were substantially superior (Figure 2f). This stark contrast underscores the recombinant platform's enhanced capacity to elicit potent nAb responses.

Aged mice exhibit unique transcriptomic profiles within their CD4⁺ T cell compartment. Following vaccination, Th1 cells in aged mice demonstrate no significant differences in enrichment pathways compared to their counterparts in young mice. The landmark discovery of “age‐differential elimination” affecting Th1 cells post‐SARS‐CoV‐2 inactivated vaccination represents a significant advancement in immunosenescence research. Critically, this phenomenon establishes that the fundamental nature of aged Th1 cells involves epigenetic silencing, rather than irreversible functional impairment; furthermore, robust antigenic stimulation can transiently release chromatin constraints.^[^ ^79^ ^]^ These findings provide direct evidence supporting the potential reversibility of immune senescence. Collectively, these insights necessitate a transition from empirical age‐stratified approaches toward mechanism‐driven precision remodeling in vaccine development, thereby establishing a scientific foundation to address global disparities in age‐related immune protection. This analysis identifies Th1 cells as a conserved response hub transcending age barriers, thereby informing the development of novel adjuvants, including Th1‐polarizing agents, designed to overcome the efficacy limitations of conventional vaccines in the elderly population. Furthermore, the identification of key immune cell signatures offers the potential for personalizing vaccination strategies to enhance overall population‐level vaccine effectiveness. Collectively, these insights contribute to a universal framework for designing vaccines against age‐associated infectious diseases such as COVID‐19, influenza, and respiratory syncytial virus. Nevertheless, rigorous clinical validation, particularly through large‐scale real‐world cohort studies in older adults, remains essential before clinical implementation.

## 4 Experimental Section

### Vaccines and Immunization

Female C57BL/6 mice, including young mice (6–8 weeks) and aged mice (16–24 months),^[^ ^80^ ^]^ were purchased from Hangzhou Ziyuan Experimental Animal Technology Co., Ltd., and Shanghai SLAC Laboratory Animal Co., Ltd. All mice were housed at the Experimental Animal Center of the First Affiliated Hospital of Zhejiang University School of Medicine. The mice were randomly assigned to groups using a random number table. All animals were maintained under pathogen‐free conditions, with a 12‐hour light/12‐hour dark cycle, a controlled temperature of ≈18–23 °C, and humidity levels of 40–60%. All experimental procedures were conducted in strict accordance with the national Guidelines for the Welfare and Ethical Review of Laboratory Animals and were approved by the Animal Ethics Committee of the First Affiliated Hospital of Zhejiang University School of Medicine (Protocol Number: 20221100A).

On day 0, Mice were subcutaneously immunized with 1 µg of SARS‐CoV‐2 inactivated vaccine/ recombinant S1 protein/ R‐Phycoerythrin protein, combined with 100 µg of aluminum adjuvant (Thermofisher, 1:1 volume ratio, total 200 µL). A booster immunization was administered on day 21 using the same dosage and method as the primary immunization. Blinding was implemented during the data detection phase. The SARS‐CoV‐2 inactivated vaccine was provided by Professor Yao Hangping's team at the First Affiliated Hospital of Zhejiang University School of Medicine (Batch No. COV202010015B).^[^ ^81^ ^]^ Euthanasia of the mice was performed via cervical dislocation.

Healthy volunteers without prior COVID‐19 vaccination were enrolled, with baseline serological specimens collected pre‐vaccination. Participants subsequently received the initial dose of SARS‐CoV‐2 inactivated vaccine(BBIBP‐CorV/CoronaVac) at local health clinics in accordance with national immunization guidelines. On Day 28 post‐initial vaccination, a follow‐up blood sample was conducted immediately before administration of the second vaccination. Written informed consent was obtained from all participants preceding study procedures, and longitudinal monitoring was maintained throughout a six‐month follow‐up period. Exclusion criteria comprised: 1) hypersensitivity to COVID‐19 vaccine components or documented vaccinal hypersensitivity reactions; 2) prior SARS‐CoV‐2 infection or current active infection; 3) established diagnosis of diabetes mellitus; 4) presence of clinically significant acute infection or acute‐phase systemic illness; 5) history of autoimmune disorders or congenital/acquired immunodeficiencies; 6) prior malignancy; 7) receipt of non‐study vaccinations within the preceding 30 days; and 8) current pregnancy. These criteria were uniformly applied to screen potential participants. This study was conducted in accordance with the ethical principles of the Declaration of Helsinki, and the study protocol was approved by the Clinical Research Ethics Committee of the First Affiliated Hospital, Zhejiang University School of Medicine (Approval No. IIT20210071A).

### SARS‐CoV‐2–Specific Antibody Measurements

All reagents were purchased from Biolegend, USA, unless otherwise mentioned. Recombinant SARS‐CoV‐2 S1protein (GenScript) was coated onto 96‐well plates at 50 µL per well (1 µg mL^−1^ in coating buffer), followed by overnight incubation at 4 °C. Subsequent to coating buffer removal and five wash cycles, plates underwent blocking for 2 hours at room temperature using 1% BSA. Serially diluted serum samples in 1% BSA were subsequently added (50 µL well^−1^) and incubated for 1 hour at room temperature. Following five additional washes, detection antibodies—biotinylated anti‐mouse IgG, IgG1, IgG2b, IgG2c (Jackson ImmunoResearch), IgG3, IgM, and IgA—were applied at 1:1000 dilution (50 µL well^−1^) with 1‐hour room temperature incubation. After further washing (five cycles), HRP‐conjugated streptavidin (Yeasen) was introduced at a 1:3000 dilution (50 µL well^−1^) for 30 min at room temperature. Post five final washes, enzymatic activity was developed using 100 µL TMB substrate (Beyotime) during 15‐min dark incubation at room temperature, with the reaction terminated by TMB stop solution (Beyotime). Absorbance measurements were recorded at 450 nm. For serum antibody affinity assessment, samples were treated with diethylamine (DEA) at a final concentration of 10 mM before analysis, with subsequent steps mirroring the antibody titer detection protocol.

The RBD‐specific IgG detection procedure strictly follows the steps outlined in the SARS‐CoV‐2 Spike Protein RBD Human IgG Enzyme‐Linked Immunosorbent Assay Kit (BD) instructions.

### Flow Cytometry

All reagents were purchased from Biolegend, USA, unless otherwise mentioned. Draining lymph nodes from vaccine‐immunized mice were collected and digested with Liberase enzyme (Roche) at a final concentration of 0.125 mg mL^−1^ and DNase I enzyme at a final concentration of 20 ug mL^−1^ at 37 ° C for 25 min. Followed by smashing with a 70‐µm strainer to make a single‐cell suspension. Before staining with fluorochrome‐conjugated antibodies, cells were blocked with Fc receptor antibody CD16/32 (clone 2.4G2, BD), 0.2 mg mL^−1^ total mouse IgG, and rat IgG on ice for 20 min.

For surface staining: Stained with fluorochrome‐conjugated antibodies in FACS buffer on ice for 30 min: PE/Cyanine5 anti‐mouse CD3(1:100 dilution), Brilliant Violet 711 anti‐mouse CXCR5(1:100 dilution), Brilliant Violet 605 anti‐mouse PD‐1(1:100 dilution), APC/Cyanine7 anti‐mouse CD4(1:100 dilution), Pacific Blue anti‐mouse CD19(1:100 dilution), PE anti‐mouse CD8a(1:100 dilution), APC anti‐mouse ICOS(1:100 dilution), APC anti‐mouse CD38(1:400 dilution), FITC anti‐mouse CD45R/B220(1:100 dilution), Brilliant Violet 711 anti‐mouse CD138(1:200 dilution), Pacific Blue anti‐mouse IgD(1:100 dilution), Brilliant Violet 650 anti‐mouse IgG1(1:100 dilution), APC/Fire 750 anti‐mouse CD62L(1:100 dilution), PE anti‐mouse CD44(1:200 dilution), PE/Cyanine7 anti‐mouse Fas(1:200 dilution), PerCP anti‐mouse CD11b(1:100 dilution), APC/Cyanine7 anti‐mouse CD11c(1:100 dilution), PE/Cyanine7 anti‐mouse F4/80(1:100 dilution), Brilliant Violet 421 anti‐mouse CD80(1:100 dilution), PE anti‐mouse CD86(1:200 dilution), AF488 anti‐mouse IAB(1:100 dilution).

For intracellular cytokine staining, cells were resuspended at 1 × 10⁷ mL^−1^ in IMDM supplemented with 10% FBS and stimulated with either: a) 1.5 µg mL^−1^ SARS‐CoV‐2 spike peptide library (JPT, PM‐WCPV‐S‐SU1‐1; N‐terminal dissolved in DMSO) plus 2.5 µg mL^−1^ anti‐mouse CD28 antibody, or b) 50 ng mL^−1^ PMA with 1 µg mL^−1^ ionomycin. Following 2‐hour incubation at 37 °C/5% CO_2_, Brefeldin A was added at a 1:1000 dilution for an additional 4‐hour incubation. Post‐stimulation, cells underwent washing with 1% FACS buffer prior to 20‐min ice incubation with Fc receptor blocking cocktail containing anti‐CD16/32 antibody, 0.2 mg mL^−1^ total mouse IgG, and rat IgG. Surface staining was subsequently performed for 30 min on ice using: Zombie Aqua Fixable Viability Kit, Pacific Blue anti‐mouse CD8α(1:100 dilution), FITC anti‐mouse CD3ε(1:100 dilution), and PerCP anti‐mouse CD4(1:100 dilution). Following washing, cells were fixed for 20 min on ice using Fixation Buffer, then permeabilized with Perm/Wash Buffer. Subsequent intracellular staining employed 30‐min ice incubation with: PE anti‐mouse IL‐21 (1:100 dilution, ThermoFisher), Brilliant Violet 711 anti‐mouse IL‐4 (1:100 dilution, BD), APC anti‐mouse IFN‐γ(1:100 dilution), and APC/Cyanine7 anti‐mouse TNF‐α(1:100 dilution). All data were acquired in BD FACS Fortessa and analyzed using FlowJo analysis software v10.

### ELISPOT

Following the addition of 15 µL of 35% ethanol to each well of a 96‐well polyvinylidene fluoride (PVDF) membrane plate and subsequent 2‐min equilibration, five washes were performed using 200 µL sterile water per well. Subsequently, 100 µL of SARS‐CoV‐2 S1 protein (1 µg/mL, GenScript) was immobilized via overnight incubation at 4 °C. After five additional sterile water washes, plates were blocked with 200 µL of 10% BSA through 1‐hour incubation at room temperature. Following five PBS washes with plate inversion for supernatant removal and gentle blotting on absorbent paper to eliminate residual buffer, freshly isolated mouse bone marrow cells were resuspended in RPMI 1640 medium supplemented with 10% NBCS. Serial dilutions of cell suspensions were then added to antigen‐coated wells and cultured for 24 hours at 37 °C/5% CO_2_. Post‐incubation, six PBS washes were performed, and then 100 µL biotin‐conjugated anti‐mouse IgG was added for 2 hours at room temperature. After six further PBS washes, alkaline phosphatase‐conjugated streptavidin (AP‐SAV, Beyotime) was applied (100 µL well^−1^) with 1‐hour incubation. Following final PBS washing, enzymatic development was initiated by adding 100 µL of freshly prepared BCIP/NBT substrate (Beyotime) according to manufacturer specifications, followed by 15‐min light‐protected incubation at room temperature. The reaction was terminated by solution removal and three distilled water washes.

### Neutralizing Antibody Assay

Serum samples were diluted with MEM cell culture medium containing 1% FBS to a final volume of 50 µL. Serial dilutions of the test serum were thoroughly mixed with an equal volume of virus suspension (200 TCID50) (WT: hCoV‐19/Hangzhou/ZJU‐05/2020, GISAID ID: EPI_ISL_415 711; Delta: hCoV‐19/Hangzhou/ZJU‐12/2021, GISAID ID: EPI_ISL_3 127 444; Omicron: hCoV‐19/Zhejiang/ZJU‐18/2022, GISAID ID: EPI_ISL_16 984 277). The mixtures were incubated at 37 °C for 2 hours. Controls were included as follows: negative antibody control (virus + medium + Vero cells), positive antibody control (virus + P17 antibody + Vero cells),^[^ ^82^ ^]^ and normal cell control (medium + Vero cells). P17 is a fully human neutralizing antibody specifically targeting the SARS‐CoV‐2 S‐RBD, which was developed by our research team through screening of a human antibody library. In this study, P17 was used as a positive control for the antibody neutralization assay.^[^ ^82^ ^]^ Following incubation, Vero cells were resuspended in MEM medium supplemented with 5% FBS and seeded into 96‐well plates containing the virus‐serum mixtures at 10⁴ cells well^−1^ in 100 µL volumes, followed by 5‐day incubation at 35 °C under humidified 5% CO_2_ atmosphere. Neutralization endpoints were then determined microscopically through assessment of cytopathic effect (CPE).

### Single Cell RNA Sequencing

Single‐cell suspensions were prepared from the draining lymph nodes of aged and young mice 14 days post‐immunization with the SARS‐CoV‐2 inactivated vaccine. Libraries were generated and sequenced from the cDNAs with Chromium Next GEM Single Cell 3′ Reagent Kits v3.1. Barcoded gel beads were combined with cell‐enzyme mixtures within microfluidic chambers to generate Gel Beads‐in‐Emulsions (GEMs), where subsequent bead dissolution released oligonucleotide capture sequences containing unique molecular identifiers (UMIs). Following reverse transcription to synthesize cDNA, template amplification via PCR constructed sequencing libraries that underwent high‐throughput paired‐end sequencing (PE150 mode) on the Illumina platform, achieving ≈50 000 reads per cell. Initial data processing and quality control were performed using Cell Ranger (v3.1.0). Briefly, reads with low‐quality barcodes and UMIs were filtered out and then mapped to the reference genome. Cells with an unusually high number of UMIs (≥8000) or mitochondrial gene percent (≥10%) were filtered out. We also excluded cells with less than 500 or more than 4000 genes detected. Additionally, doublet GEMs should also be filtered out. It was achieved by using the tool DoubletFinder (v2.0.3). After removing low‐quality cells, we utilized Harmony for data integration and batch effect correction. The integrated data underwent PCA dimensionality reduction, followed by clustering of the reduced‐dimensionality data using the soft k‐means clustering algorithm. This approach probabilistically assigns cells to clusters, maximizing dataset diversity within each cluster. The global centroid for all datasets within each cluster, as well as dataset‐specific centroids, were computed. Correction factors were then calculated for each dataset to adjust cells toward these centroids. This process was iterated until the clustering results stabilized. The clusters were manually annotated based on established cell subtype marker genes. Differential gene expression analysis between experimental groups employed the Wilcoxon rank‐sum test, while significantly enriched KEGG pathways (Q≤0.05) were identified through hypergeometric testing and multiple‐testing correction. Pseudotemporal trajectory reconstruction was implemented in Monocle2, with all analytical workflows executed in R version 3.6.3. All generated mouse scRNAseq data in this study have been deposited into the Genome Sequence Archive (GSA) with accession number (GSA: CRA030892).

### IFN‐γ Neutralization Assay

Following random allocation to control and experimental cohorts, all mice received subcutaneous immunization with the SARS‐CoV‐2 inactivated vaccine on Day 0. The experimental group underwent intraperitoneal administration of anti‐mouse IFN‐γ monoclonal antibody (10 mg kg^−1^) (Bioxcell) at Days 0, 3, and 6 post‐immunization, while control animals received equivalent volumes of PBS at identical timepoints. Terminal blood collection was performed on Day 7 for subsequent quantification of S1‐specific antibody titers in serum.

### CD4^+^ T Cell Adoptive Transfer Assay

For CD4⁺ T cell depletion, 6–8‐week CD45.1⁺ C57BL/6 mice received intraperitoneal administration of 200 µg anti‐mouse CD4 monoclonal antibody (clone GK1.5, Bioxcell). 1 week post‐depletion, 1 × 10⁷ purified and enriched CD4⁺ T cells from either aged or young CD45.2⁺ C57BL/6 donor mice were adoptively transferred into recipient mice via tail vein injection. The enrichment of CD4^+^ T cells was performed by using the CD4^+^ T cell enrichment kit according to the manufacturer's instructions, while control cohorts received isovolumetric PBS. The next day, all recipient mice underwent subcutaneous immunization with the SARS‐CoV‐2 inactivated vaccine. Terminal collection of blood and draining lymph nodes was performed 7 days post‐immunization for concurrent assessment of serum anti‐S1 IgG titers and draining lymph node immunophenotyping.

### Statistical Analysis

Data analysis was performed using SPSS (version 25.0) and R (version 3.6.3). Graphs were generated with GraphPad Prism (version 9.0) and Origin software (version 9.8.0). For comparisons between two groups of normally distributed data, an unpaired two‐tailed Student's t‐test was applied; for non‐normally distributed data, the Mann–Whitney U test was used. Correlations between variables were evaluated using Pearson's correlation coefficient for normally distributed data and Spearman's rank correlation coefficient for non‐normally distributed data. Continuous data are presented as mean ± standard deviation (SD) for normally distributed variables or as median with interquartile range (IQR) for non‐normally distributed variables. Statistical significance was denoted as follows: *p < 0.05, **p < 0.01, ***p < 0.001, and NS indicated no significant difference between groups.

## Conflict of Interest

The authors declare no conflict of interest.

## Author Contributions

H.Y., X.Z., and Y.Y. designed the project; C.Y. was involved in experimental assays throughout the study, analyzed the data, and wrote the manuscript. X.Z., J.G., and Y.L. conducted prospective population cohort data collection and contributed to clinical data acquisition along with antigen‐specific antibody detection. L.Q., J.C., and Z.M. assisted in the establishment of animal models and performed the collection of tissue specimens and serum samples. H.J. and J.L. participated in the discussion. The paper was edited by all co‐authors.

## Supporting information



Supplemental Figure 1‐9

Supplemental Table 1

Supplemental Table 2

## Data Availability

The source data for the main figures in this study have been deposited in Zenodo and are publicly available. The dataset can be accessed via the following persistent DOI: https://doi.org/10.5281/zenodo.17379410. The data that support the findings of this study are openly available in Zenodo at https://doi.org/10.5281/zenodo.17379410, reference number [17379410].
