# Supplementary material for: Pre‐Existing Th1 Immunity Outperforms Age in Predicting Antibody Responses to SARS‐CoV‐2 Inactivated Vaccines
Source: Adv Sci (Weinh). 2025 Nov 16;13(6):e14147. doi: 10.1002/advs.202514147 (PMC12866872; doi:10.1002/advs.202514147)
Supplement: Supplementary file 1 — Supplemental Figure 1‐9 [file ADVS-13-e14147-s001.pdf]

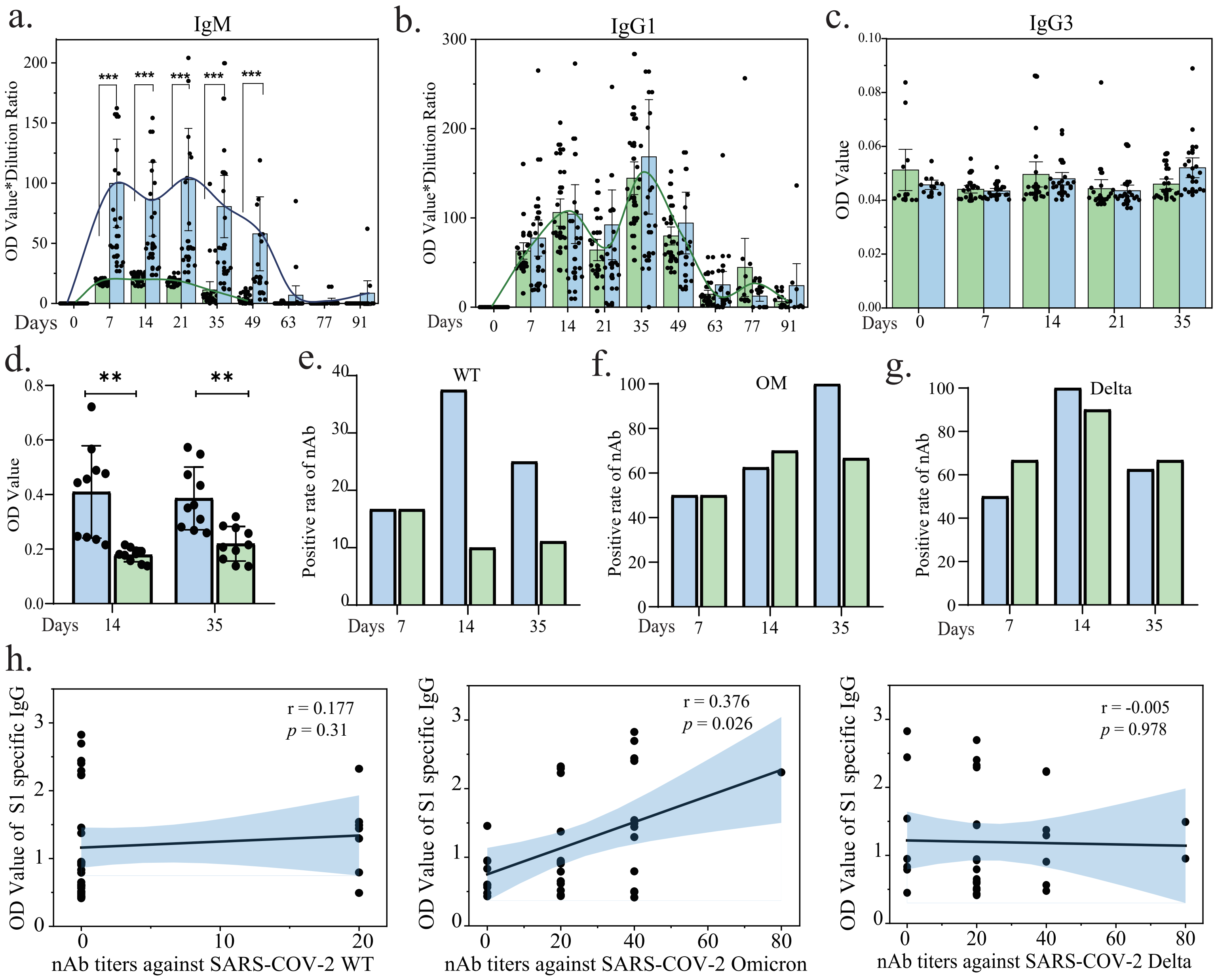
a.
IgM
OD Value*Dilution Ratio
***
Days 0 7 14 21 35 49 63 77 91
b.
IgG1
OD Value*Dilution Ratio
Days 0 7 14 21 35 49 63 77 91
c.
IgG3
OD Value
Days 0 7 14 21 35
d.
**
OD Value
Days 14 35
e.
WT
Positive rate of nAb
Days 7 14 35
f.
OM
Positive rate of nAb
Days 7 14 35
g.
Delta
Positive rate of nAb
Days 7 14 35
h.
r = 0.177
p = 0.31
OD Value of S1 specific IgG
nAb titers against SARS-COV-2 WT
r = 0.376
p = 0.026
OD Value of S1 specific IgG
nAb titers against SARS-COV-2 Omicron
r = -0.005
p = 0.978
OD Value of S1 specific IgG
nAb titers against SARS-COV-2 Delta

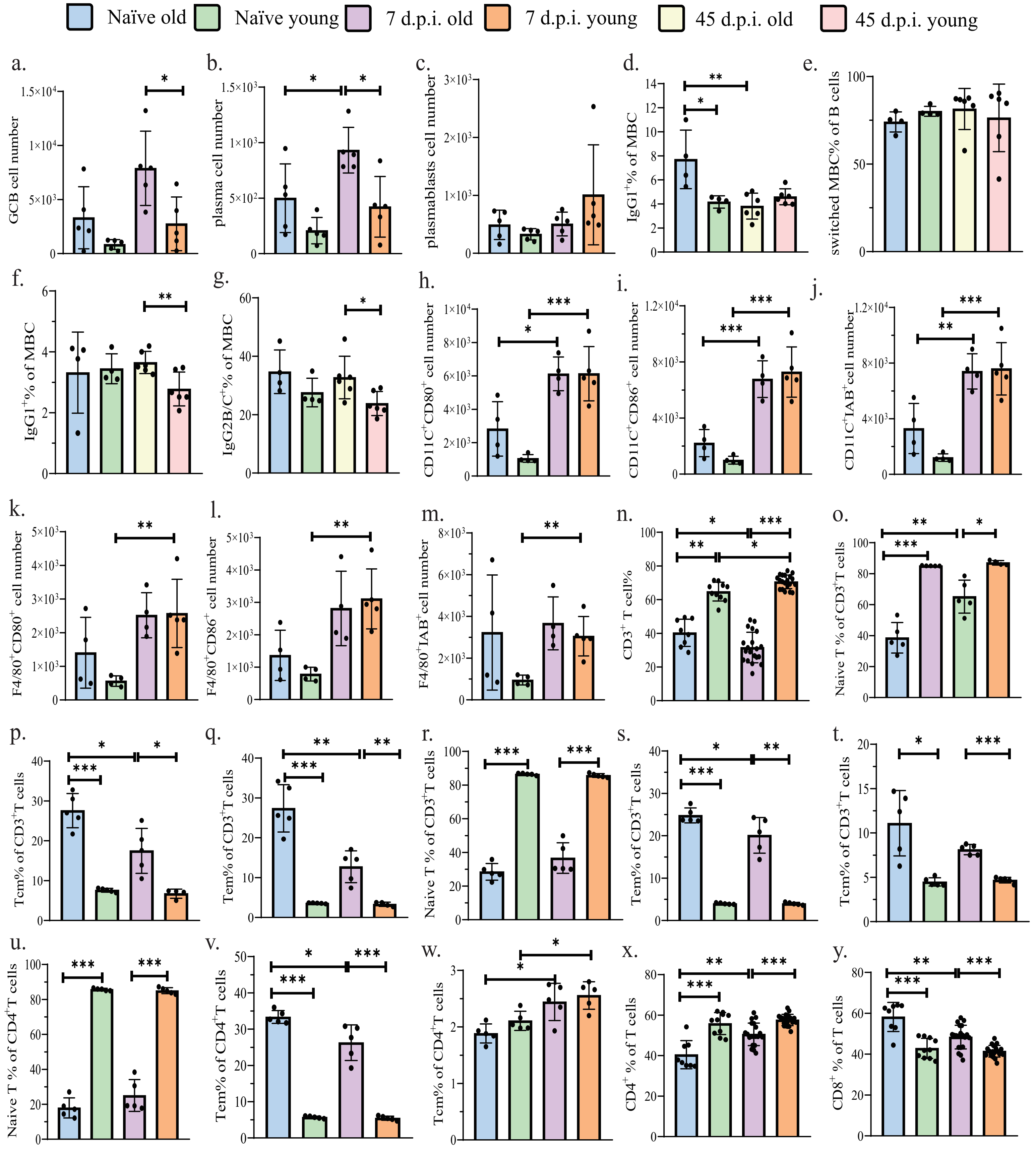

Naïve old
Naïve young
7 d.p.i. old
7 d.p.i. young
45 d.p.i. old
45 d.p.i. young
a.
GCB cell number
b.
plasma cell number
c.
plasmablasts cell number
d.
IgG1+% of MBC
e.
switched MBC% of B cells
f.
IgG1+% of MBC
g.
IgG2B/C+% of MBC
h.
CD11C+CD80+ cell number
i.
CD11C+CD86+ cell number
j.
CD11C+IAB+cell number
k.
F4/80+CD80+ cell number
l.
F4/80+CD86+ cell number
m.
F4/80+IAB+cell number
n.
CD3+ T cell%
o.
Naive T % of CD3+T cells
p.
Tcm% of CD3+T cells
q.
Tem% of CD3+T cells
r.
Naive T % of CD3+T cells
s.
Tem% of CD3+T cells
t.
Tcm% of CD3+T cells
u.
Naive T % of CD4+T cells
v.
Tem% of CD4+T cells
w.
Tcm% of CD4+T cells
x.
CD4+ % of T cells
y.
CD8+ % of T cells

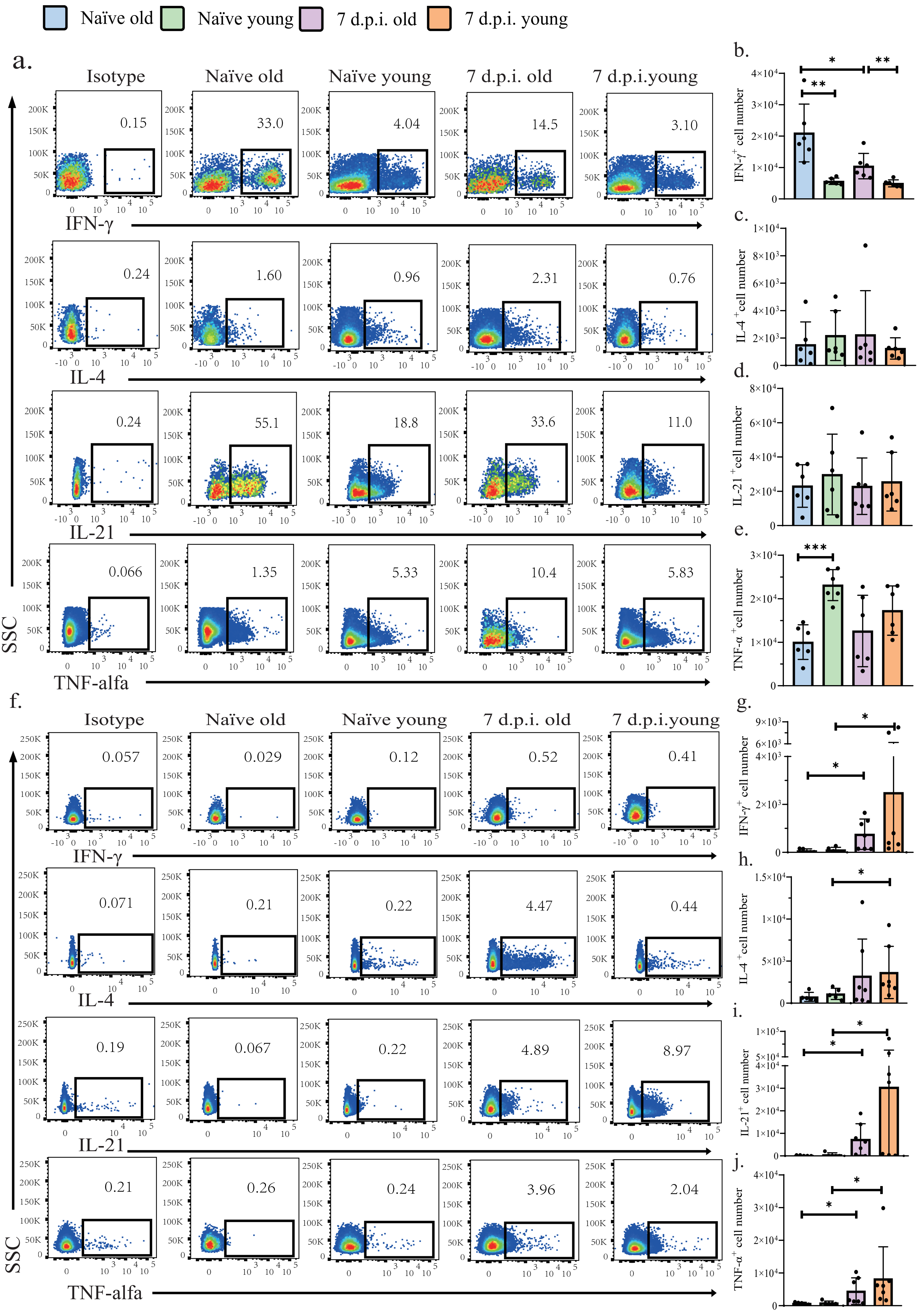

Naïve old
Naïve young
7 d.p.i. old
7 d.p.i. young
a.
Isotype
Naïve old
Naïve young
7 d.p.i. old
7 d.p.i.young
0.15
33.0
4.04
14.5
3.10
IFN-γ
0.24
1.60
0.96
2.31
0.76
IL-4
0.24
55.1
18.8
33.6
11.0
IL-21
0.066
1.35
5.33
10.4
5.83
TNF-alfa
SSC
b.
IFN-γ+ cell number
c.
IL-4 + cell number
d.
IL-21 + cell number
e.
TNF-α + cell number
f.
Isotype
Naïve old
Naïve young
7 d.p.i. old
7 d.p.i.young
0.057
0.029
0.12
0.52
0.41
IFN-γ
0.071
0.21
0.22
4.47
0.44
IL-4
0.19
0.067
0.22
4.89
8.97
IL-21
0.21
0.26
0.24
3.96
2.04
TNF-alfa
SSC
g.
IFN-γ+ cell number
h.
IL-4 + cell number
i.
IL-21+ cell number
j.
TNF-α+ cell number

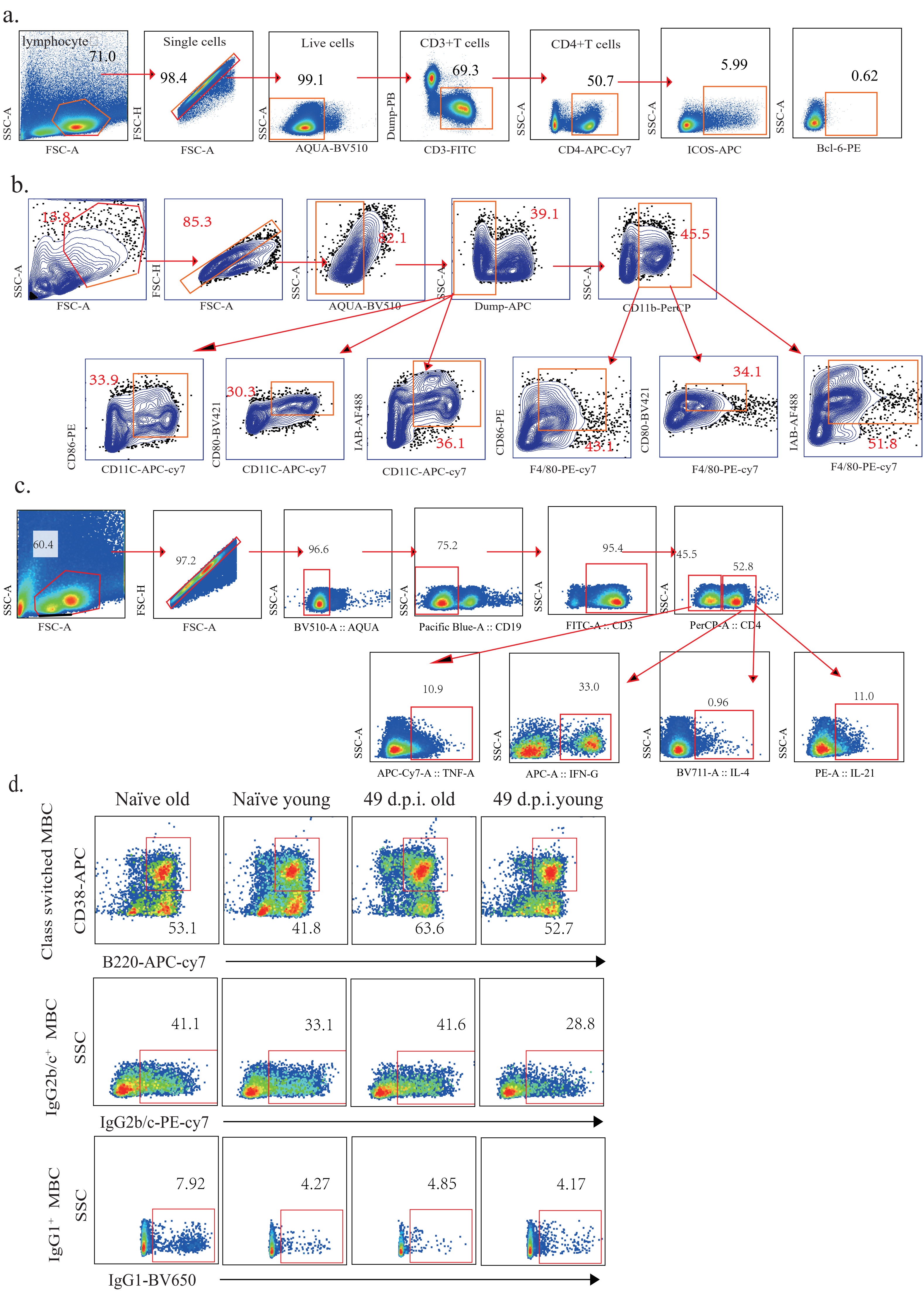
a.
lymphocyte
71.0
Single cells
98.4
Live cells
99.1
CD3+T cells
69.3
CD4+T cells
50.7
5.99
0.62
SSC-A
FSC-A
FSC-H
AQUA-BV510
Dump-PB
CD3-FITC
CD4-APC-Cy7
ICOS-APC
Bcl-6-PE
b.
13.8
85.3
82.1
39.1
45.5
Dump-APC
CD11b-PerCP
33.9
30.3
36.1
43.1
34.1
51.8
CD86-PE
CD80-BV421
IAB-AF488
CD11C-APC-cy7
F4/80-PE-cy7
c.
60.4
97.2
96.6
75.2
95.4
45.5
52.8
BV510-A :: AQUA
Pacific Blue-A :: CD19
FITC-A :: CD3
PerCP-A :: CD4
10.9
33.0
0.96
11.0
APC-Cy7-A :: TNF-A
APC-A :: IFN-G
BV711-A :: IL-4
PE-A :: IL-21
d.
Naïve old
Naïve young
49 d.p.i. old
49 d.p.i.young
Class switched MBC
CD38-APC
53.1
41.8
63.6
52.7
B220-APC-cy7
IgG2b/c+ MBC
SSC
41.1
33.1
41.6
28.8
IgG2b/c-PE-cy7
IgG1+ MBC
7.92
4.27
4.85
4.17
IgG1-BV650

a.

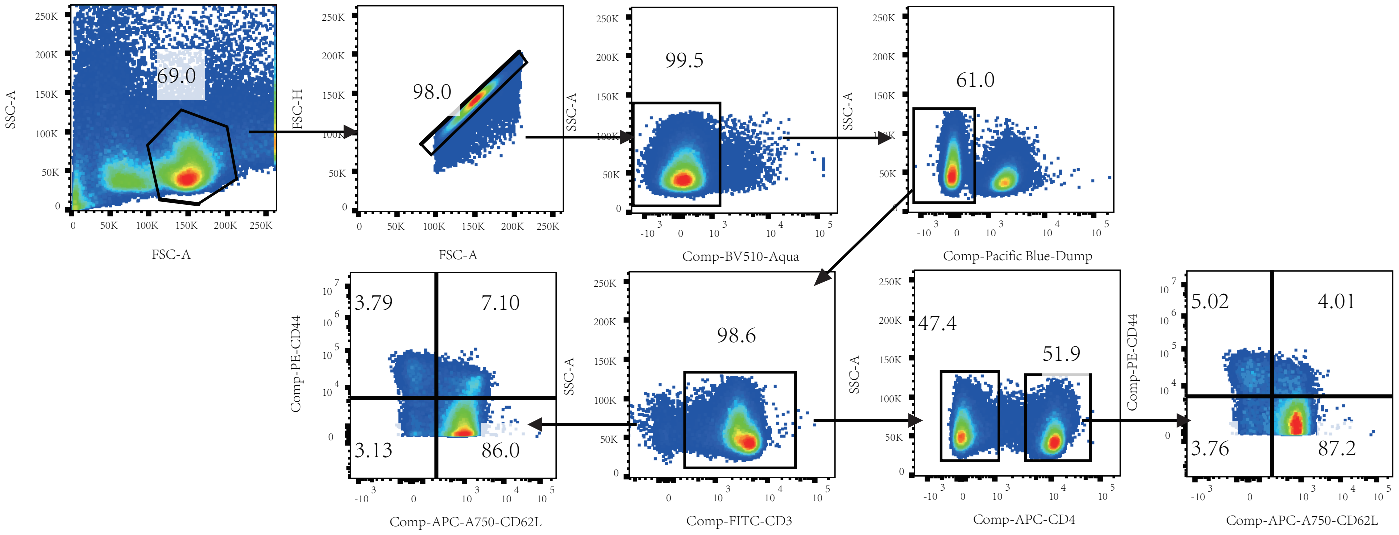
69.0
98.0
99.5
61.0
SSC-A
FSC-H
FSC-A
Comp-BV510-Aqua
Comp-Pacific Blue-Dump
3.79
7.10
3.13
86.0
98.6
47.4
51.9
5.02
4.01
3.76
87.2
Comp-PE-CD44
Comp-APC-A750-CD62L
Comp-FITC-CD3
Comp-APC-CD4

b.

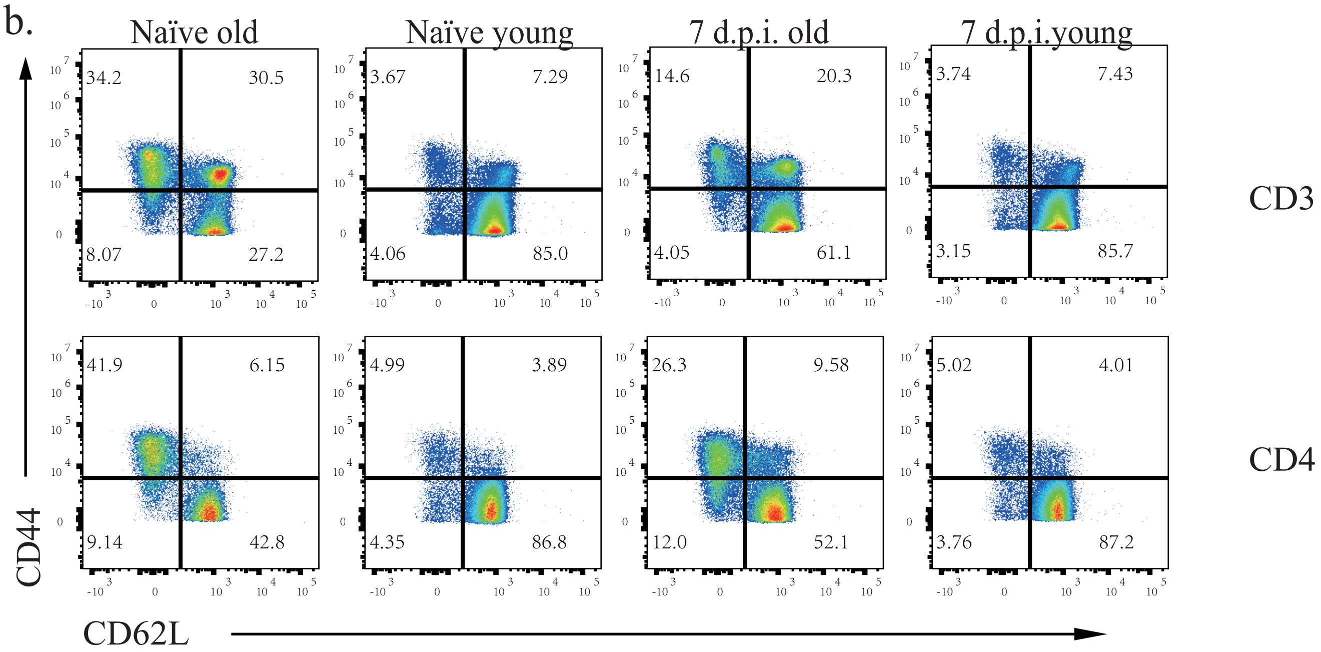
Naïve old
Naïve young
7 d.p.i. old
7 d.p.i.young
34.2
30.5
8.07
27.2
3.67
7.29
4.06
85.0
14.6
20.3
4.05
61.1
3.74
7.43
3.15
85.7
CD3
41.9
6.15
9.14
42.8
4.99
3.89
4.35
86.8
26.3
9.58
12.0
52.1
5.02
4.01
3.76
87.2
CD4
CD44
CD62L

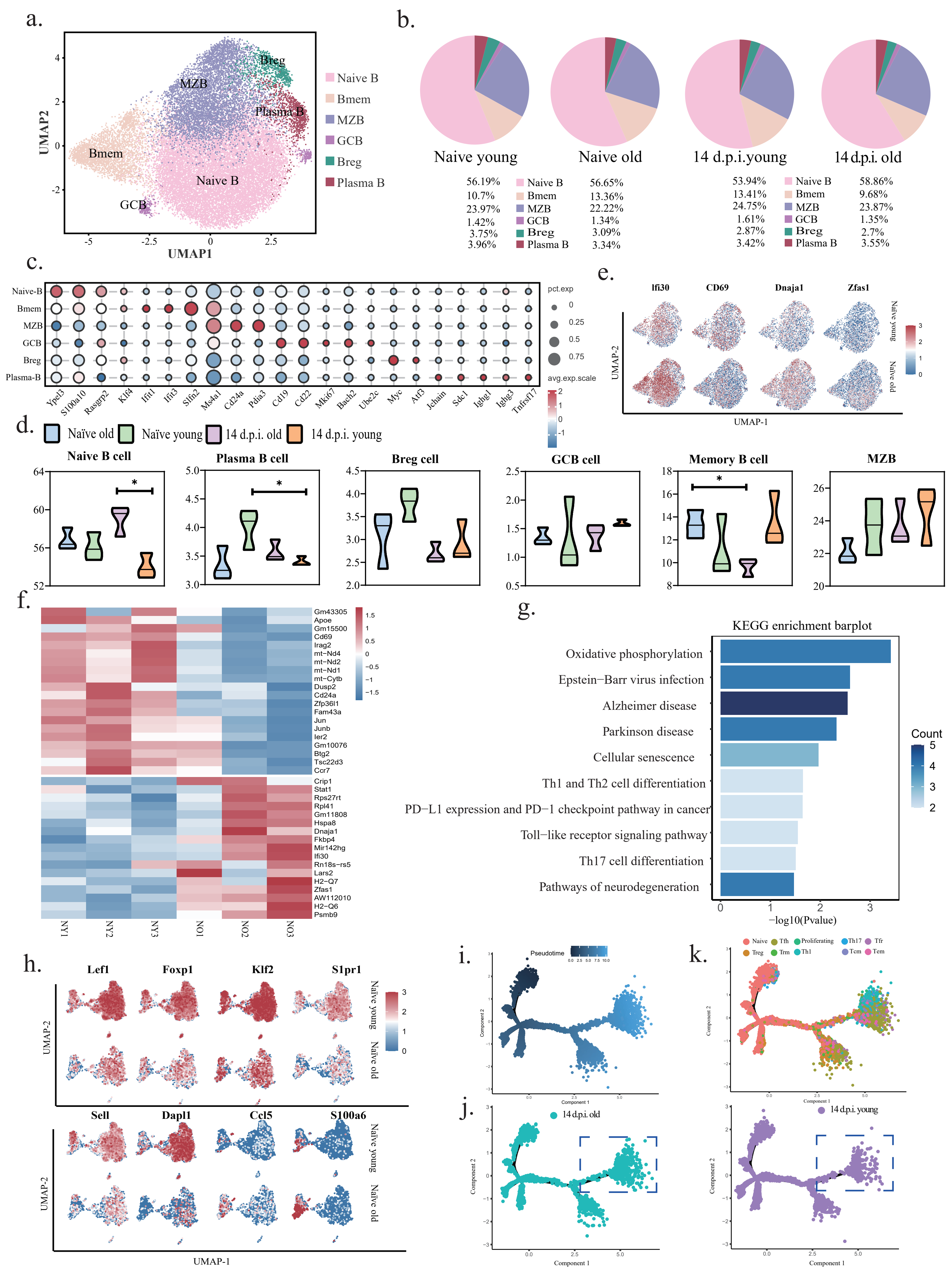

a.
UMAP2
UMAP1
Breg
MZB
Plasma B
Bmem
Naive B
GCB
Naive B
Bmem
MZB
GCB
Breg
Plasma B
b.
Naive young
Naive old
14 d.p.i.young
14 d.p.i. old
56.19%
10.7%
23.97%
1.42%
3.75%
3.96%
Naive B
Bmem
MZB
GCB
Breg
Plasma B
56.65%
13.36%
22.22%
1.34%
3.09%
3.34%
53.94%
13.41%
24.75%
1.61%
2.87%
3.42%
Naive B
Bmem
MZB
GCB
Breg
Plasma B
58.86%
9.68%
23.87%
1.35%
2.7%
3.55%
c.
Naive-B
Bmem
MZB
GCB
Breg
Plasma-B
Ypel3
S100a10
Rasgrp2
Klf4
Ifit1
Ifit3
Slfn2
Ms4a1
Cd24a
Pdia3
Cd19
Cd22
Mki67
Bach2
Ube2c
Myc
Atf3
Jchain
Sdc1
Ighg1
Ighg3
Tnfrsf17
pct.exp
avg.exp.scale
e.
Ifi30
CD69
Dnaja1
Zfas1
Naïve young
Naïve old
UMAP-2
UMAP-1
d.
Naïve old
Naïve young
14 d.p.i. old
14 d.p.i. young
Naive B cell
Plasma B cell
Breg cell
GCB cell
Memory B cell
MZB
f.
Gm43305
Apoe
Gm15500
Cd69
Irag2
mt-Nd4
mt-Nd2
mt-Nd1
mt-Cytb
Dusp2
Cd24a
Zfp36l1
Fam43a
Jun
Junb
Ier2
Gm10076
Btg2
Tsc22d3
Ccr7
Crip1
Stat1
Rps27rt
Rpl41
Gm11808
Hspa8
Dnaja1
Fkbp4
Mir142hg
Ifi30
Rn18s-rs5
Lars2
H2-Q7
Zfas1
AW112010
H2-Q6
Psmb9
NY1
NY2
NY3
NO1
NO2
NO3
g.
KEGG enrichment barplot
Oxidative phosphorylation
Epstein−Barr virus infection
Alzheimer disease
Parkinson disease
Cellular senescence
Th1 and Th2 cell differentiation
PD−L1 expression and PD−1 checkpoint pathway in cancer
Toll−like receptor signaling pathway
Th17 cell differentiation
Pathways of neurodegeneration
Count
−log10(Pvalue)
h.
Lef1
Foxp1
Klf2
S1pr1
Sell
Dapl1
Ccl5
S100a6
Naïve young
Naïve old
UMAP-2
UMAP-1
i.
Pseudotime
Component 1
Component 2
k.
Naive
Tfh
Proliferating
Th17
Tfr
Treg
Trm
Th1
Tem
Tcm
j.
14 d.p.i. old
14 d.p.i. young

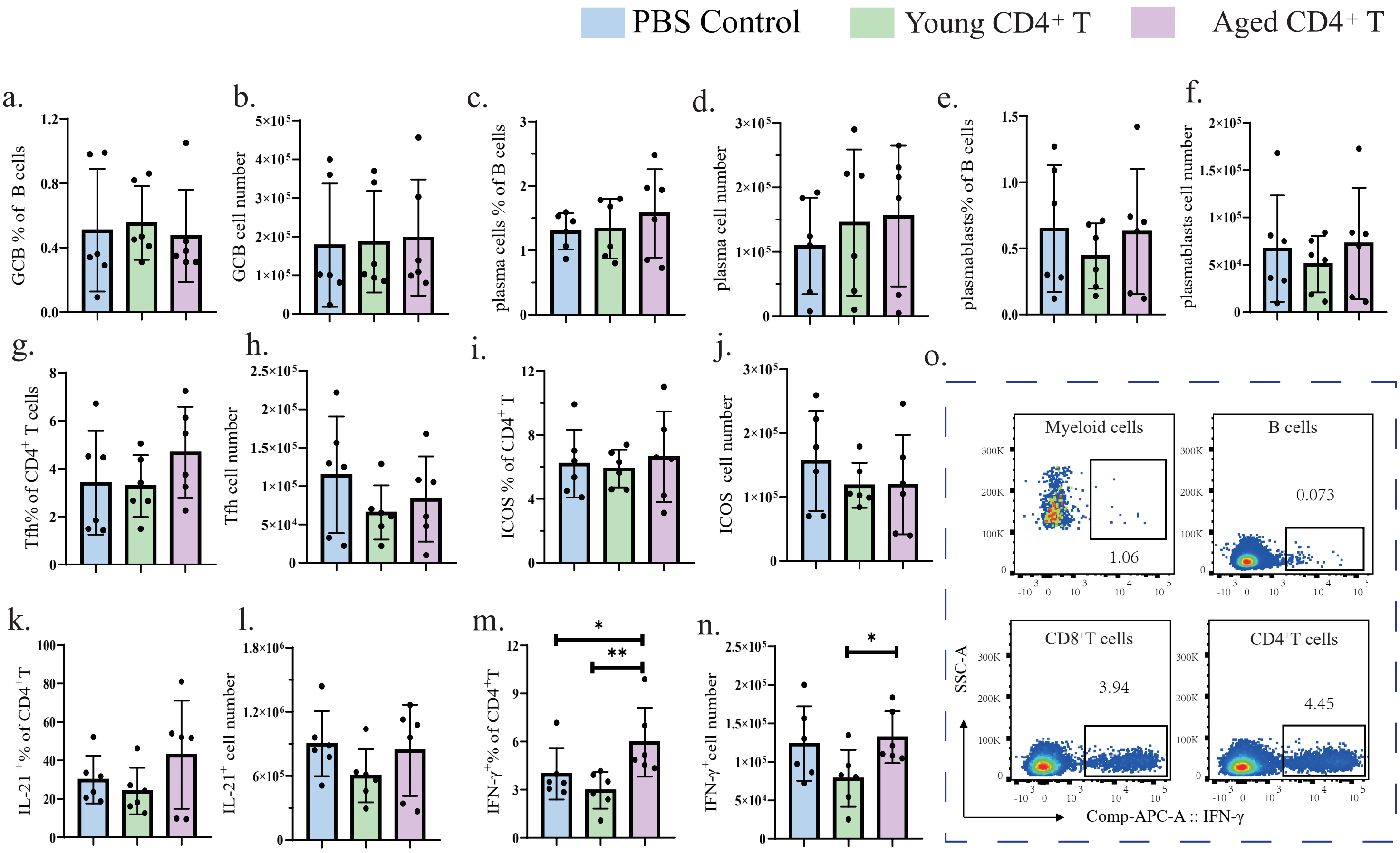
PBS Control
Young CD4+ T
Aged CD4+ T
a.
GCB % of B cells
b.
GCB cell number
c.
plasma cells % of B cells
d.
plasma cell number
e.
plasmablasts% of B cells
f.
plasmablasts cell number
g.
Tfh% of CD4+ T cells
h.
Tfh cell number
i.
ICOS % of CD4+ T
j.
ICOS cell number
k.
IL-21+% of CD4+T
l.
IL-21+ cell number
m.
IFN-γ+% of CD4+T
*
**
n.
IFN-γ+cell number
*
o.
Myeloid cells
1.06
B cells
0.073
CD8+T cells
3.94
CD4+T cells
4.45
SSC-A
Comp-APC-A :: IFN-γ

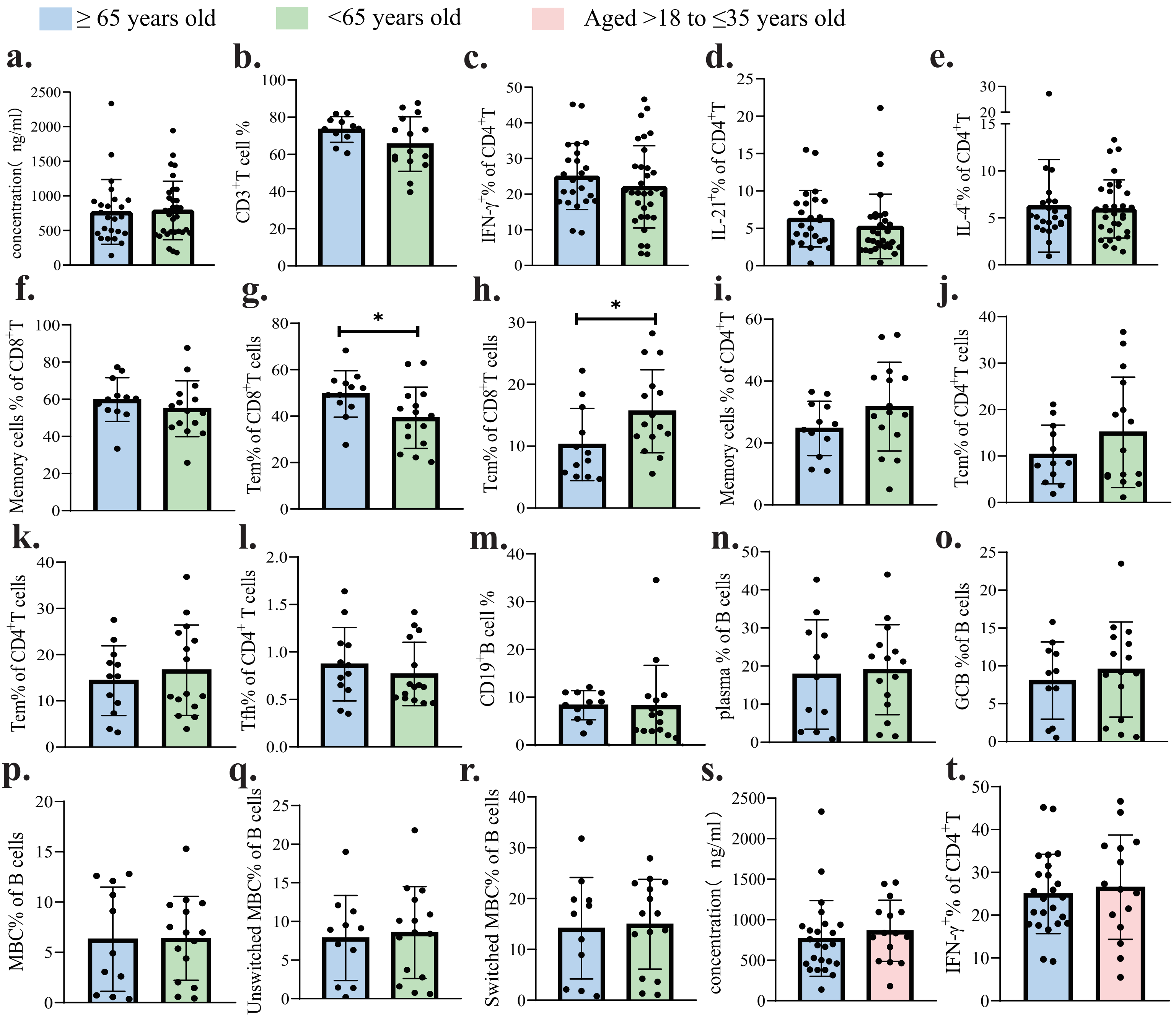
≥ 65 years old
<65 years old
Aged >18 to ≤35 years old
a.
concentration(ng/ml)
b.
CD3+T cell %
c.
IFN-γ+% of CD4+T
d.
IL-21+% of CD4+T
e.
IL-4+% of CD4+T
f.
Memory cells % of CD8+T
g.
Tem% of CD8+T cells
*
h.
Tcm% of CD8+T cells
*
i.
Memory cells % of CD4+T
j.
Tcm% of CD4+T cells
k.
Tem% of CD4+T cells
l.
Tfh% of CD4+ T cells
m.
CD19+B cell %
n.
plasma % of B cells
o.
GCB %of B cells
p.
MBC% of B cells
q.
Unswitched MBC% of B cells
r.
Switched MBC% of B cells
s.
concentration(ng/ml)
t.
IFN-γ+% of CD4+T

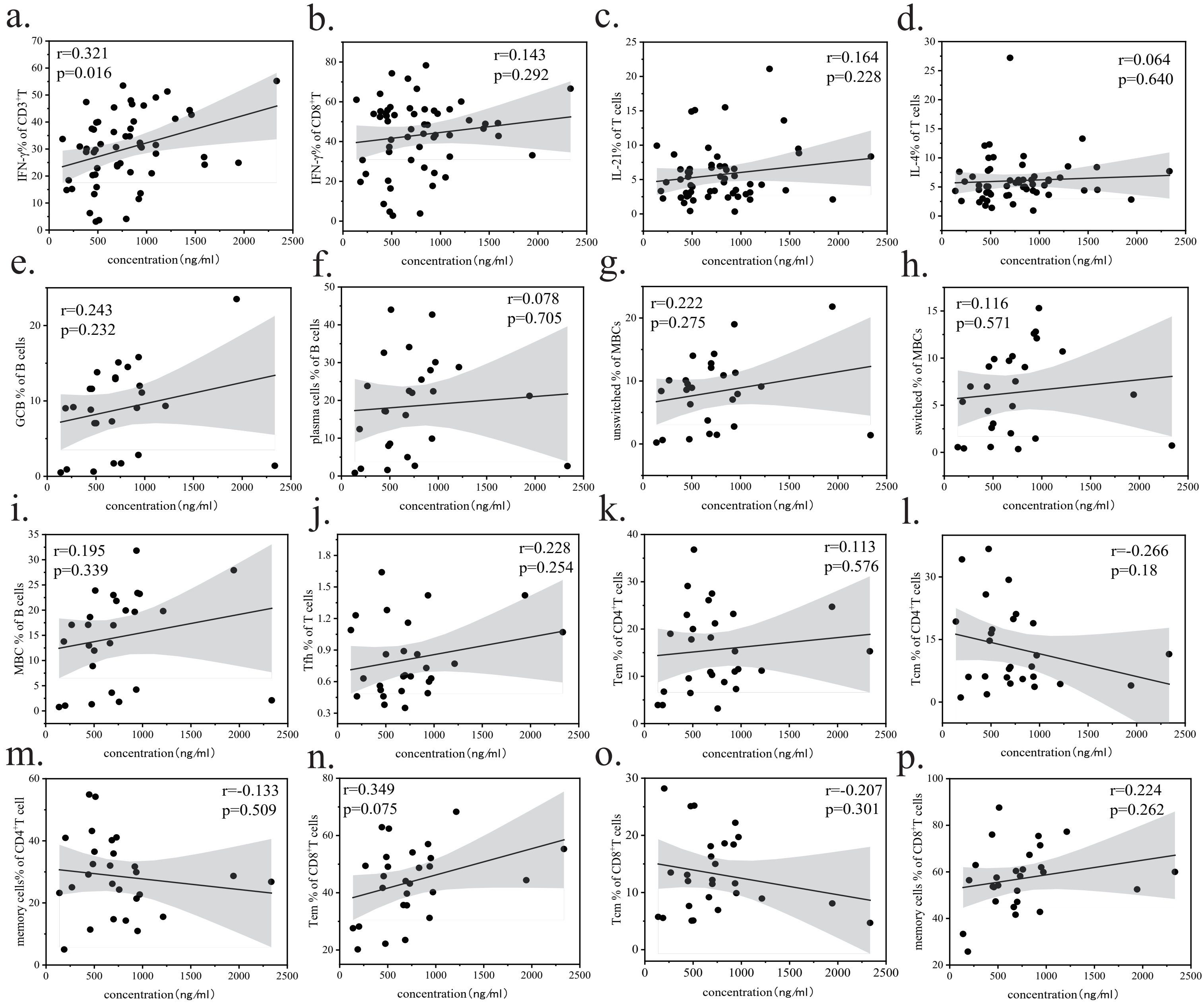
a.
r=0.321
p=0.016
IFN-γ% of CD3+T
concentration (ng/ml)
b.
r=0.143
p=0.292
IFN-γ% of CD8+T
concentration (ng/ml)
c.
r=0.164
p=0.228
IL-21% of T cells
concentration (ng/ml)
d.
r=0.064
p=0.640
IL-4% of T cells
concentration (ng/ml)
e.
r=0.243
p=0.232
GCB % of B cells
concentration (ng/ml)
f.
r=0.078
p=0.705
plasma cells % of B cells
concentration (ng/ml)
g.
r=0.222
p=0.275
unswitched % of MBCs
concentration (ng/ml)
h.
r=0.116
p=0.571
switched % of MBCs
concentration (ng/ml)
i.
r=0.195
p=0.339
MBC % of B cells
concentration (ng/ml)
j.
r=0.228
p=0.254
Tfh % of T cells
concentration (ng/ml)
k.
r=0.113
p=0.576
Tem % of CD4+T cells
concentration (ng/ml)
l.
r=-0.266
p=0.18
Tcm % of CD4+T cells
concentration (ng/ml)
m.
r=-0.133
p=0.509
memory cells% of CD4+T cell
concentration (ng/ml)
n.
r=0.349
p=0.075
Tem % of CD8+T cells
concentration (ng/ml)
o.
r=-0.207
p=0.301
Tcm % of CD8+T cells
concentration (ng/ml)
p.
r=0.224
p=0.262
memory cells % of CD8+T cells
concentration (ng/ml)
