# Supplementary material for: Pre‐Existing Th1 Immunity Outperforms Age in Predicting Antibody Responses to SARS‐CoV‐2 Inactivated Vaccines
Source: Adv Sci (Weinh). 2025 Nov 16;13(6):e14147. doi: 10.1002/advs.202514147 (PMC12866872; doi:10.1002/advs.202514147)
Supplement: Supplementary file 2 — Supplemental Table 1 [file ADVS-13-e14147-s003.docx]

Table S1.Baseline clinical characteristics of the study cohort

|  | **Aged（≥65 years）** | **Young(18 to <65 years)** |  |
| --- | --- | --- | --- |
|  | **n=24** | **n=32** | ***P* Value** |
| **Age** | 69（67-71） | 40（31-60） | <0.001 |
| **Female** | 11(45.8%) | 8(25.0%) | 0.103 |
| **Male** | 13(54.2%) | 24(75.0%) | 0.103 |
| **Hypertension** | 16(66.6%) | 3(9.4%) | <0.001 |
| **Diabetes mellitus** | 0(0.0%) | 0(0.0%) | / |
| **autoimmune disorders** | 0(0.0%) | 0(0.0%) | / |
| **congenital/acquired immunodeficiencies** | 0(0.0%) | 0(0.0%) | / |
| **malignancy** | 0(0.0%) | 0(0.0%) | / |
| **Chronic liver disease** | 0(0.0%) | 0(0.0%) | / |
| **Other comorbidities** | 0(0.0%) | 0(0.0%) | / |
| **SARS-CoV-2–specific antibody(ng/ml)** | 693.4(458.5-933.3) | 757.9(474.3-1031.5) | 0.722 |
