# Supplementary material for: Pre‐Existing Th1 Immunity Outperforms Age in Predicting Antibody Responses to SARS‐CoV‐2 Inactivated Vaccines
Source: Adv Sci (Weinh). 2025 Nov 16;13(6):e14147. doi: 10.1002/advs.202514147 (PMC12866872; doi:10.1002/advs.202514147)
Supplement: Supplementary file 3 — Supplemental Table 2 [file ADVS-13-e14147-s002.docx]

Table S2. Multivariable Gamma Regression Analysis of Factors Associated with Antibody Titer (n=56)

| **Variable** | **β Coefficient** | **Standard Error** | **Exp(β)** | **95% CI** | **P Value** |
| --- | --- | --- | --- | --- | --- |
| **Demographic Factors** |  |  |  |  |  |
| Age(year) | 0.003 | 0.005 | 1.003 | (-0.007, 0.013) | 0.534 |
| Sex(Female vs Male) | 0.002 | 0.143 | 1.002 | (-0.277, 0.281) | 0.989 |
| **Clinical Factors** |  |  |  |  |  |
| Hypertension (Yes vs No) | 0.267 | 0.181 | 1.306 | (-0.088, 0.622) | 0.141 |
| **Main Predictor** |  |  |  |  |  |
| Baseline Th1 level | 0.022 | 0.006 | 1.022 | (0.010, 0.035) | <0.001 |
| **Intercept** | 5.764 | 0.439 | 312.899 | (4.903, 6.624) | <0.001 |
